# Interactive Analysis, Exploration, and Visualization of RNA-Seq Data with SeqCVIBE

**DOI:** 10.3390/mps5020027

**Published:** 2022-03-18

**Authors:** Efthimios Bothos, Pantelis Hatzis, Panagiotis Moulos

**Affiliations:** 1 Institute of Communications and Computer Systems, National Technical University of Athens, 15780 Athens, Greece; mpthim@mail.ntua.gr; 2 HybridStat Predictive Analytics PC, Evrota 25, 14564 Kifisia, Greece; 3 Institute for Fundamental Biomedical Research, Biomedical Sciences Research Center Alexander Fleming, Fleming 34, 16672 Vari, Greece; hatzis@fleming.gr

**Keywords:** RNA-sequencing, differential expression, data visualization, Shiny, gene expression, big data

## Abstract

The rise of modern gene expression profiling techniques, such as RNA-Seq, has generated a wealth of high-quality datasets spanning all fields of current biological research. The large data sets and the continually expanding applications for which they can be mined, such as the investigation of alternative splicing and others, have created novel challenges for data management, exploration, analysis, and visualization. Although a large variety of RNA-Seq data analysis software packages has emerged, both open-source and commercial, most fail to simultaneously address the above challenges, while they lack obvious functionalities, such as estimating RNA abundance over non-annotated genomic regions of interest in real time. We have developed SeqCVIBE, an R Shiny web application for the interactive exploration, analysis, visualization, and genome browsing of large RNA-Seq datasets. SeqCVIBE allows for multiple on-the-fly visualizations and calculations, such as differential expression analysis, averaging genomic signals over specific regions of the genome, and calculating RNA abundances over custom, potentially non-annotated regions, such as novel long non-coding RNAs. In addition, SeqCVIBE comprises a database for pre-analyzed data, where users can navigate and explore results, as well as perform a variety of basic on-the-fly analyses and export the outcomes. Finally, we demonstrate the value of SeqCVIBE in the elucidation of the interplay of a novel lincRNA, WiNTRLINC1, and Wnt signaling in colon cancer.

## 1. Introduction

Among the many applications of RNA-Seq, such as de novo transcriptome assembly [1], allele specific gene expression associations [2], evidence on transcript fusion [3], and many others [4], Differential Gene Expression Analysis (DGEA) of gene expression profiles appears to still attract the majority of interest. RNA-Seq data and the associated DGEA not only provide detailed, intuitive, and comprehensive results on genome-wide expression profiles, but also a wealth of additional insights, such as rich visualizations of the underlying RNA signal patterns and structures as well as quantification of previously undetected or non-annotated transcribed genomic regions [5]. In addition, with the emergence of single-cell transcriptomics technologies [6] and their unlimited portfolio of applications regarding the investigation of biological mechanisms at the single-cell level, the popularity of DGEA appears to continuously grow, while being challenged towards the development of new methods adapted to the different nature of single-cell high-throughput transcriptomics.

Driven by the popularity of RNA-Seq, serious research effort has been devoted to the development of dedicated DGEA methods, spanning all the processing steps, from raw data quality control, quantification, and normalization, up to the derivation of differentially expressed genes and further downstream analyses including ontology and pathway enrichment [7]. At the same time, DGEA algorithms attempt to address some of the inherent biases of RNA-Seq, such as the gene-length bias (read accumulation over longer transcripts), which propagates to DGEA and the instability of the latter with respect to different normalization frameworks [8], or the under-representation of certain transcript types, such as lncRNAs [9].

To tackle these problems, we previously developed PANDORA, a weighted p-value combination algorithm for DGEA [8,10]. By combining the individual p-values of RNA-Seq data-specific statistical tests using a weighting scheme derived from simulations based on real data, PANDORA optimizes DGEA by ensuring the best precision-sensitivity relationship. Furthermore, PANDORA appears to be insusceptible to biases introduced by different normalization frameworks, offers improved DGEA when applied to low count RNA molecules, such as lncRNAs, and is able to control for the propagation of gene length bias in downstream functional and enrichment analysis [8]. PANDORA is implemented in the Bioconductor packages metaseqR and metaseqR2, which offer an interface for several normalization, DGEA, and p-value combination methods, along with interactive analysis reports with quality control metrics and visual representation of the results. However, these reports are static in the sense that users cannot query data or create new visualizations dynamically.

Another direct result of the increasing popularity of RNA-Seq, both at the bulk as well as the single-cell level, is the unprecedented increase in the availability of generally good-quality datasets. This exponential increase is provisioned by the range of biological questions posed to gene expression data, the continuous addition of new research protocols, and the decreasing sequencing costs [11]. In most cases, research-related (and less often, clinic-related) gene expression data are stored in data warehouses and repositories of various volumes, encompassing datasets with various experimental complexities and sample sizes. This already fuzzy landscape is further complicated by the variety in data release policies, especially with respect to human data or data derived from private sector studies, which often live in scarce repositories and are dominated by privacy rules different in each country and organization. Therefore, the quality of the vast volumes of generated data becomes further questionable, since there is a lack in quality control transparency and clear harmonized policies for data deposition.

There are mostly two categories of RNA-Seq data repositories. The first category encompasses raw data repositories, such as the Gene Expression Omnibus (GEO) and the European Nucleotides Archive (ENA). Such repositories are restricted to hosting raw data in a structured manner along with basic dataset (or series in GEO terms) and sample descriptions, which allow easy retrieval while offering very limited exploration and analysis capabilities and almost non-existent quality control. The second category is more loosely defined and includes mostly pre-analyzed and partly curated gene expression data, often co-existing with other -omics data layers (e.g., proteomics, DNA methylation, etc.) and centered around specific disease or tissue/organ themes (expression atlases). A typical example of a disease focused -omics data repository is The Cancer Genome Atlas project (https://portal.gdc.cancer.gov/, accessed on 11 March 2022), which comprises >20,000 molecularly characterized cancer cases, including gene expression data spanning 33 cancer types [12]. The TCGA portal offers (authorized) access to raw data as well as exploration and various visualizations of processed and pre-analyzed data at various -omic layers. A typical example of a tissue focused -omics data repository is the The Genotype-Tissue Expression (GTEx) portal (https://gtexportal.org/ accessed on 11 March 2022). GTEx is an ongoing project [13] offering access to gene expression and regulation data across several human tissues and organs (54 tissues, >15,000 samples). While both the TCGA and GTEx repositories offer a great user experience with respect to data navigation and exploration of basic results and generalized visualizations, users cannot go deeper in sample exploration. Thus, they are not able to inspect RNA-Seq signals in a genome browser or perform any analyses on-the-fly. The latter are either very restricted or essentially limited in building dynamic queries for data retrieval and display basic statistics. The only way to perform more complex analyses coupled with more advanced visualizations is to retrieve the data of interest to be analyzed by field experts, such as trained bioinformaticians.

It is therefore evident that modern RNA-Seq data also suffer from a recent phenomenon observed with most high-throughput technologies [14]: data production rates overwhelm data management, computing, and analytics capabilities. This unceasingly creates further challenges, both for scientists trying to mine meaningful results of potential clinical importance and analysts and software engineers, who are challenged to organize and harmonize the huge data volumes. In order to prototype a combinatorial data management and analysis system, as well as to enhance PANDORA as an analytical tool for RNA-Seq data, we developed SeqCVIBE, a web application for the interactive exploration, analysis, visualization, and genome browsing of RNA-Seq datasets. Apart from offering a rich data visualization suite, SeqCVIBE allows for several on-the-fly calculations, such as averaging and visualizing genomic tracks over specific genomic regions and calculating RNA abundances in custom regions, such as novel long non-coding RNAs. In addition, SeqCVIBE can be configured as a database for pre-analyzed data, where the user can navigate and explore results, as well as perform a variety of basic on-the-fly analyses and export the outcomes. Apart from the platform implementation and functionalities, we also present a series of usage scenarios that demonstrate SeqCVIBE’s utility in facilitating purposes from basic and advanced exploration of RNA-Seq datasets to education. A demo SeqCVIBE instance is available within the Hypatia cloud infrastructure developed within the frameworks of the Elixir-GR project at http://elixir-seqcvibe.hybridstat.gr (accessed on 15 March 2022). The codebase of SeqCVIBE and deployment instructions can be found at https://github.com/hybridstat/seqcvibe (accessed on 15 March 2022).

## 2. Materials and Methods

### 2.1. Dataset Retrieval and Basic Quality Control

For dataset retrieval for the GEO, we used the SRA toolkit, version 2.11.0 (https://github.com/ncbi/sra-tools, accessed on 11 March 2022) and the program’s prefetch to fetch the respective SRA archives and fastq-dump to extract the FASTQ files from the SRA archives. For dataset retrieval from ENA, we used the fastq-dl (https://github.com/rpetit3/fastq-dl, accessed on 11 March 2022) program which implements ENA’s API for raw data retrieval. After FASTQ extraction, we used FastQC, version 0.11.8 (https://www.bioinformatics.babraham.ac.uk/projects/fastqc/, accessed on 11 March 2022) to acquire fundamental quality control metrics. Low quality samples that could not become usable after trimming 5′ or 3′ ends of the reads were removed. For the rest, we used seqtk 1.3 (https://github.com/lh3/seqtk, accessed on 11 March 2022) to trim the reads where required. FastQC raw text results were used with MultiQC to create the interactive reports available through SeqCVIBE.

### 2.2. RNA-Seq Data Preprocessing

The quality controlled FASTQ files were subjected to alignment to the reference genomes using a two-way alignment procedure to achieve better successful alignment rates. Firstly, RNA-Seq reads were aligned with the splice-aware aligner hisat2 2.2.1 [15]. The unmapped reads were subjected to a second alignment round using Bowtie2 [16] with the --local --very-sensitive-local switches on. The process was adjusted accordingly for datasets with single and paired-end reads respectively. The human datasets were aligned against the human reference genome version hg19 (GRCh37) and the mouse datasets to the mouse reference genome mm10 (GRCm38). The resulting BAM files were processed with metaseqR2 in order to produce read count tables (raw and normalized with DESeq) and normalized genome browser visualization tracks. Regarding noise filtering, we did not directly hard-filter the read counts. Instead, we attached flags to each gene/transcript to allow user-based custom filtering within SeqCVIBE later. The preprocessing results were stored in R data files (rda) and placed properly with respect to SeqCVIBE data storage structure, as described in the Implementation section. A brief description of the preprocessing process can be found in the repository https://github.com/hybridstat/elixir-gr-project, accessed on 15 March 2022.

## 3. Implementation

### 3.1. Overview of SeqCVIBE

**Figure 1:**
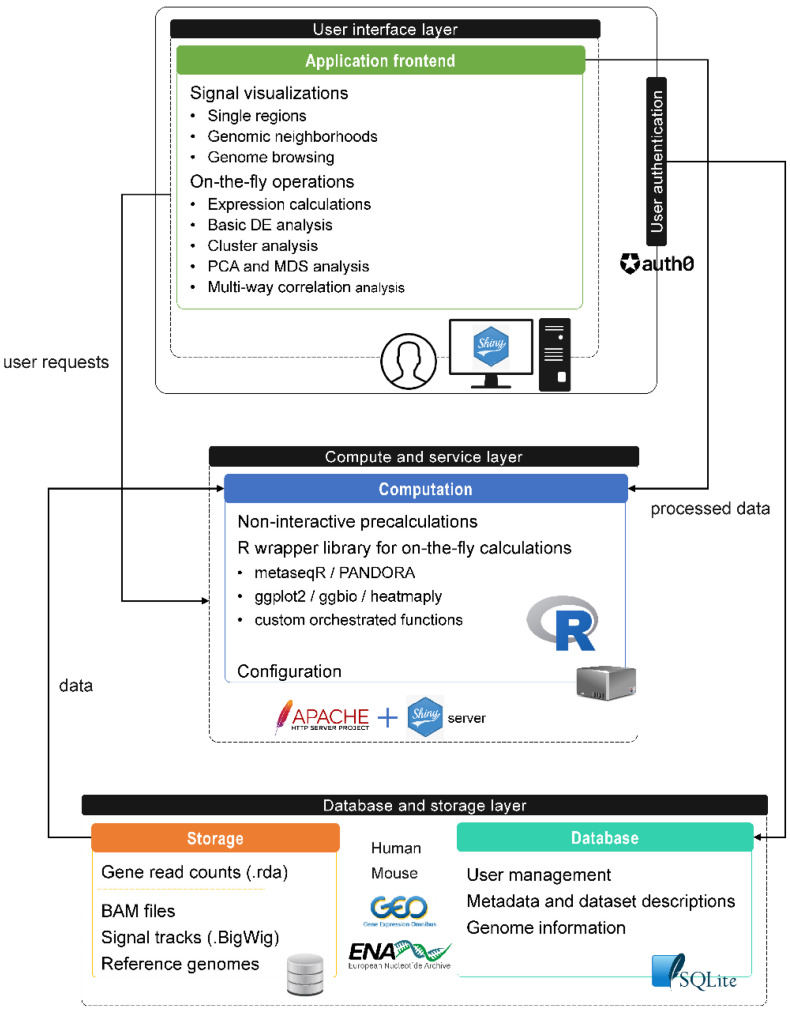
The different layers of SeqCVIBE (database and storage, compute and service, user interface) with their components, a summary of their functionalities, and the communication between them.

SeqCVIBE is a web application developed using the R Shiny framework, a web development framework for the R statistical language, which is quickly gaining popularity among data scientists, including bioinformaticians. The application consists of three distinct layers (Figure 1). Within the database and storage layer, an SQLite database hosts application metadata required for proper data management. The compute and service layer hosts an R function library which is responsible for (i) preprocessing, formatting, and harmonizing of data imported and visualized in SeqCVIBE and (ii) performing several on-the-fly calculations requested by the user in real-time, such as expression abundances calculated directly from BAM files over possibly expressed but non-annotated regions, such long non-coding RNAs. The Shiny server is responsible for the web function of SeqCVIBE while being served over Apache for scale and compatibility with other visualization components, such as the JBrowse genome browser and the display of RNA-Seq signal tracks.

The database and storage layer includes the static storage components which host a specific file structure for each dataset hosted in SeqCVIBE. This dataset-wise structure briefly includes:

1. Quality control reports, generated with MultiQC [17].
2. Optionally aligned and spliced reads for each sample (BAM files).
3. Normalized RNA-Seq signal tracks (BigWig files).
4. Precalculated read counts for each exon of each annotated gene/transcript of the reference genome used for read alignment.
5. Genomic co-ordinate annotation files for each gene of the reference genome used for read alignment.

The processing steps required for items (2)–(4) are described in detail in Materials and Methods. BAM and BigWig files as well as precalculated read counts are categorized according to the experimental design (biological conditions and samples) present in the metadata structure of each hosted dataset. Apart from RNA-Seq data, the storage layer includes the required reference genomes in formats suitable for displaying on the JBrowse genome browser. Additional reference genome information and metadata accompanying each processed RNA-Seq dataset, along with user and session management information, are stored in a simple SQLite database. Finally, the user interface layer includes the integrated application frontend (Shiny application and JBrowse), fortified by user authentication processes managed by the external service auth0 (www.auth0.com, accessed on 11 March 2022) and a related R package developed for communication of Shiny with auth0.

### 3.2. Application Structure

The operations supported by SeqCVIBE span a variety of purposes, from administrative tasks including user and session management, to various RNA-Seq analytics and visualizations, including signal averaging visualization and abundance estimation over known and non-annotated genomic regions, basic DGEA, and other tools for querying gene expression profiles. These tools include Principal Component Analysis (PCA) and MultiDimensional Scaling (MDS), clustering analysis, profile correlations, etc., as well as genome browsing. The following subsections briefly describe SeqCVIBE’s features. It should be noted that all the subsequently mentioned tables and figures are exportable in several formats through the respective application controls.

#### 3.2.1. Administrative Functions

SeqCVIBE supports basic user and data administration functionalities, such as user registration and user credential management (username, email, password) as well as login and logout functionalities. The basic user management is performed through the external specialized service auth0. SeqCVIBE also supports user session management functionalities categorized as follows:

- Data sample set management, through the navigation and selection among datasets and samples hosted in SeqCVIBE and the subsequent creation of dataset instances for analysis and querying with the selected biological conditions and samples. Dataset instances can be edited and deleted.
- Analysis instance management, through the ability to work on specific dataset instances, stores the steps as Shiny bookmarks and restores the analytical steps at later times to continue work. Analysis instances can be edited and deleted.

**Figure 2:**
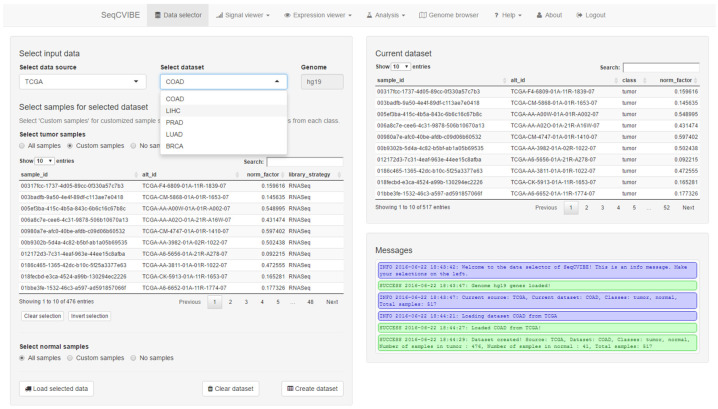
Simple dataset and sample set management functionalities in SeqCVIBE. Users can create, edit, delete, and load pre-analyzed RNA-Seq datasets imported in the SeqCVIBE database with simple backend scripts. Datasets can be subsetted to study issues such as dataset subgrouping as related to differential gene expression signatures and quality controls. Informative messages are displayed on the bottom right to inform the users regarding the data management process.

At most steps, SeqCVIBE also informs the users about actions performed or actions to be potentially taken, through the display of messages in each situation. Finally, MultiQC reports are available for each RNA-Seq dataset. A snapshot of the administrative functions is depicted in Figure 2.

#### 3.2.2. Dynamic RNA-Seq Signal Plots

**Figure 3:**
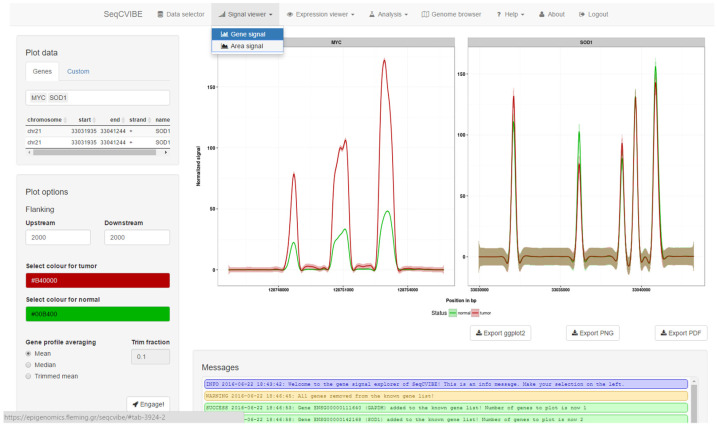
RNA-Seq signal dynamic plots. With SeqCVIBE, RNA-Seq signal visualization is achieved simply by defining the genes or genomic areas of interest either by name or by genomic coordinates and using the respective button controls to generate the plots. Figures can be exported in various formats, including a ggplot2 object for further manipulation by expert users. Track-like figures are also available through the integration of the ggbio Bioconductor package. RNA-Seq signal averaging and visualization on-the-fly are among the unique features of SeqCVIBE.

SeqCVIBE offers interactive and real-time exploration of averaged RNA-Seq signal coverage profiles generated by aligned reads across samples for many genes or custom genomic regions. The signals are automatically grouped per biological condition (depicted by different colors) and faceted plots are generated per gene or genomic region of interest. A snapshot of dynamic RNA-Seq signal plots is depicted in Figure 3. More specifically, SeqCVIBE can generate average coverage plots for:

- Genomic regions within and around known annotated transcripts.
- Genomic regions within and around non-annotated regions where transcription events have been detected via RNA-Seq, such as potentially novel long non-coding RNAs.
- Larger genomic regions within the same chromosome, with the goal of exploring average signals from multiple areas at the same time. This feature resembles the functionalities of a genome browser, but the tracks are averaged, providing a better overview, especially in the case of datasets with many samples. This is achieved with functionalities from the ggbio Bioconductor package [18].

#### 3.2.3. RNA Abundance Calculations

**Figure 4:**
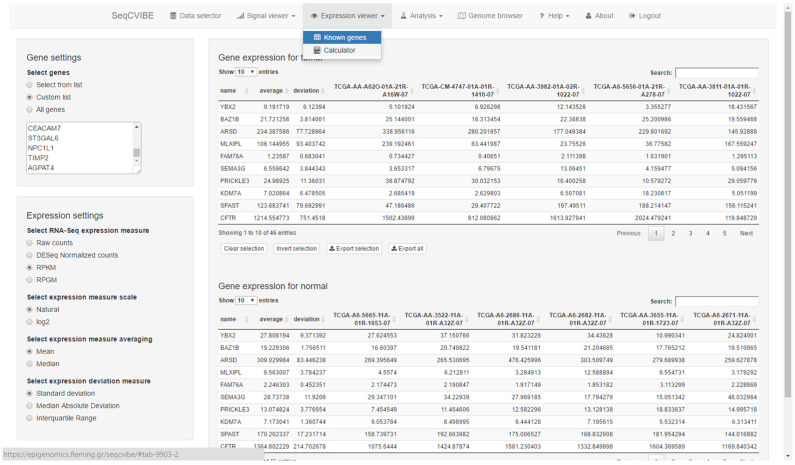
RNA-Seq abundance calculations. Users can calculate and explore RNA-Seq gene expression signal values using various transformations for all conditions and replicates in the imported datasets. Genes can be looked up by simply pasting gene names or selecting from lists. Selections can be made for exporting in text tab-delimited formats. One of the unique features of SeqCVIBE is the on-the-fly calculation of RNA-Seq signal over non-annotated areas (e.g., novel lncRNAs) by just providing the genomic coordinates.

The platform supports the interactive presentation of RNA abundance values for known genes as well as custom genomic regions. An example of RNA abundance calculations is presented in Figure 4. More specifically, SeqCVIBE supports the abilities to:

- Display gene expression values for known genes and annotated regions at various scales and summary metrics (raw, normalized, logarithmic, RPKM, etc.).
- Calculate abundances on-the-fly for non-annotated genomic regions. This functionality requires the presence of BAM files.

#### 3.2.4. Real-Time DGEA and Exploration

**Figure 5:**
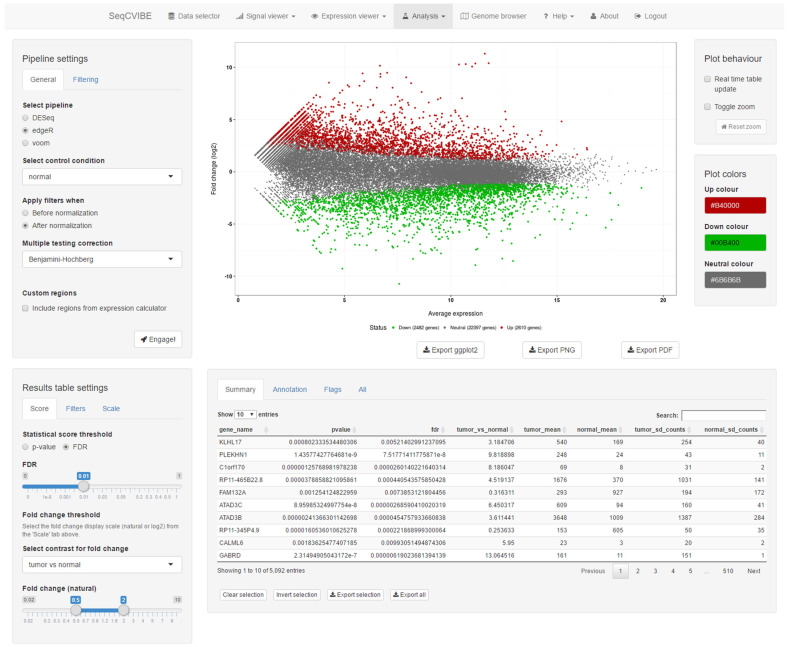
Basic DGEA. DGEA is possible on-the-fly with SeqCVIBE by deploying a lite version of the metaseqR Bioconductor data, which provides an interface to popular statistical analysis methods. Visualization and filtering of the results is performed interactively and reactively. Figures are interactive and can be exported in various formats, including a ggplot2 object for further manipulation by expert users. Tables are exported in text tab-delimited format.

Along with customized RNA signal visualizations, SeqCVIBE offers basic DGEA on user-created dataset instances through the integration of the metaseqR package and basic controls through a graphical interface. It should be noted that not all methods that ship with metaseqR are available for real-time DGEA, as some (e.g., NOISeq and baySeq) require substantially more time to run than, for example, edgeR. After the completion of DGEA, the results are displayed in interactive plots and tables, where genes of interest can be selected and highlighted. A snapshot of DGEA capabilities is provided in Figure 5. More specifically, the DGEA integrated with SeqCVIBE includes:

- Joint analysis with several statistical methods available with metaseqR, reporting joint statistical outcomes.
- Visualization of the DGEA results with respect to statistical and differential abundance outcomes through an interactive MA plot which can be used to select and filter outcomes in real-time, along with displaying in table format.
- Various interactive filters for the selection of gene sets of interest, along with the display of gene expression values in multiple arithmetic scales.

#### 3.2.5. Additional Expression Analysis Tools

**Figure 6:**
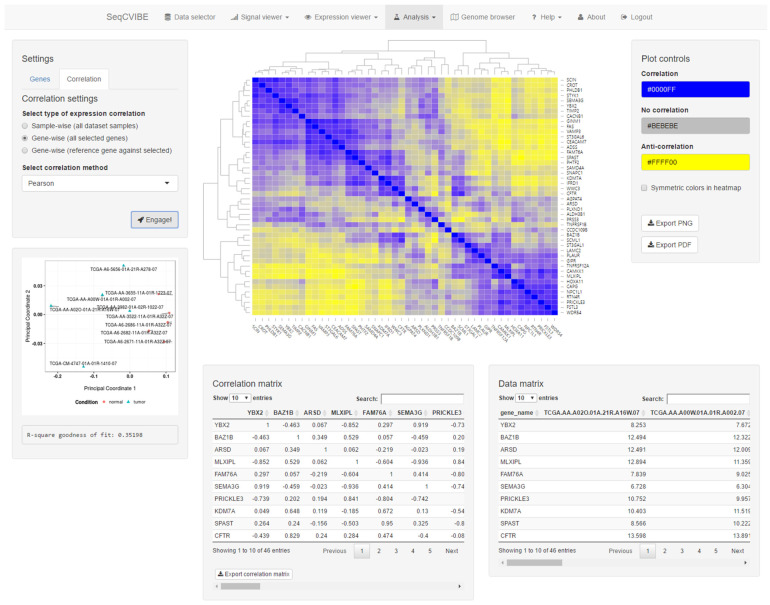
Interactive correlation analysis. SeqCVIBE offers facilities to perform gene correlation analysis using several metrics and visualizations (interactive heatmap and multi-dimensional scaling plots). Genes can be correlated in pairs (two genes over a set of biological conditions, such as a time-course experiment) or pairwise using calculating correlation matrices and related visualizations. The related gene expression is summarized in the bottom right table and tables and figures can be exported.

Apart from signal visualization and DGEA, SeqCVIBE offers a suite of tools widely used in gene expression analysis. Specifically, it supports hierarchical clustering with a variety of algorithms and settings, along with interactive displays of the resulting heatmaps and the ability to export diagrams in various formats. In addition, the platform offers a rich option set for correlation analysis of gene expression and pairwise correlations of all expressed or user-selected genes, using expression values in different supported arithmetic scales and correlation methods. Correlation analyses can be performed gene-wise, i.e., as correlation between two or more genes using all samples in the dataset instance, or sample-wise, i.e., as correlation between two or more samples from the dataset instance using all selected genes (expressed, user selected, etc.). A snapshot of correlation analysis and visuals is depicted in Figure 6. All correlation analyses are automatically visualized with the following tables and diagrams:

- Heatmap with the correlation values, also including hierarchical clustering for the estimation of sample similarities and the detection of potential outliers.
- Interactive tables with the correlation values and the expression values of the genes that are part of the correlation analysis.
- Real-time MDS plot using the genes and samples being analyzed, as additional means for estimating sample quality and outlier detection.

**Figure 7:**
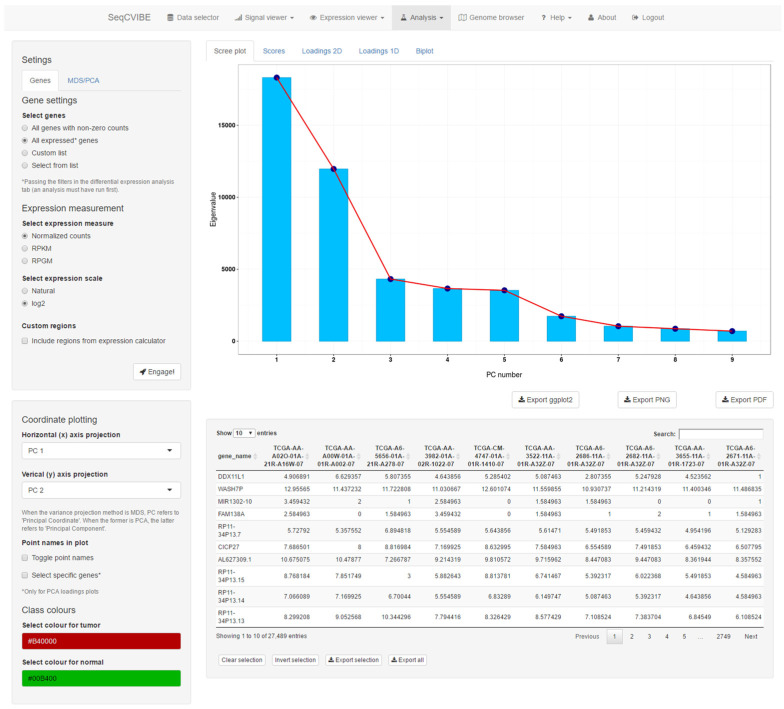
PCA functionality. SeqCVIBE provides a rich visualization suite for principal component analysis, which can be performed on various transformations of gene expression values. The results can be visualized in interactive scree, scores, loadings (2D, 1D), and biplot plots. Figures can be exported in various formats.

Furthermore, SeqCVIBE supports the exploration of dimensionality reduction through PCA and MDS. The latter can be performed using various geometrical distances and the number of eigenvectors for the construction of principal coordinates, ending in the respective interactive diagrams. The same apply for PCA, but a richer visualization suite is offered, including PCA scores and loading plots across one and two dimensions and PCA biplots (scatterplots in the reduced PCA space, where also the original dimension directions are depicted). An example of PCA-related visuals is presented in Figure 7. In both MDS and PCA, the graphs can be configured by selecting the available principal components for PCA or coordinates for MDS, while the expression of the genes analyzed is available in table format within the dimensionality reduction interface.

#### 3.2.6. Genome Browsing

**Figure 8:**
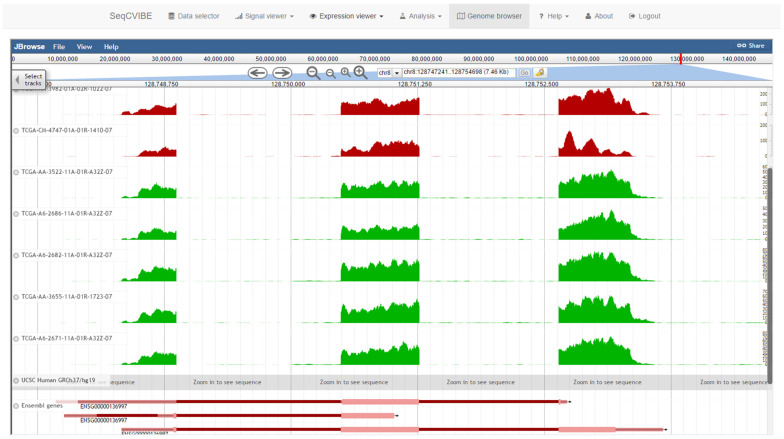
Integrated genome browser. Genome browsing of the imported dataset is made possible through the integration of the JBrowse embeddable genome browser. Starting from BAM files, all other calculations are made automatically (including normalization of BigWig signals) and the JBrowse configuration files are also automatically created and updated. The different track colors represent distinct biological conditions.

Genome browsing is an invaluable tool for the detailed visualization of gene expression through the mapping of RNA-Seq signals and usually comprises the first point of contact of the researcher interrogating the data. It is also widely accepted that NGS signal tracks often synthetize the ground truth and offer a sanity check for any subsequent analyses. SeqCVIBE integrates JBrowse [19], a popular portable genome browser which is well-supported and suitable for integration in 3rd party web applications. Signal tracks are organized per dataset and colored per biological condition. The supported transcript reference tracks include Ensembl, RefSeq, and UCSC gene annotations. A snapshot of the functionality is provided in Figure 8.

## 4. Results

### 4.1. Usage Scenarios

Based on user needs mostly related to the on-the-fly analysis and comprehensive real-time RNA signal visualization both for annotated and non-annotated genomic regions, we developed SeqCVIBE, an interactive platform for RNA-Seq data management, exploration, and visualization. The main motivation behind development was to facilitate simple RNA-Seq data management, from data import, preprocessing, and simple operations, such as big dataset subsetting by non-experts, up to comprehensive summarization and visualization of RNA-Seq signals and basic DGEA. The latter should be possible without endless navigation on genome browsers, unfriendly spreadsheets, and multiple software tools. In order to further clarify our motivation, we describe three basic usage scenarios, spanning research and education.

*Usage scenario 1: My team has evidence on a novel long non-coding RNA with potential therapeutic properties in various cancer types. I want to check whether its expression affects nearby genes in my experimental as well as public data.*

This scenario involves RNA-Seq data generated and owned by the lab that underlies the new discovery as well as public RNA-Seq data retrieved from public repositories, or controlled access repositories such as TCGA, after the respective access applications and granted permissions. The lab data may be derived from engineered human cell lines in the form of a knock-out design, where the expression of the putative novel lncRNA is restricted and compared to a wild-type cell line. The public data may be derived from a human cancerous cell line from the same tissue of origin and the experimental design may involve the monitoring of gene expression after drug administration. This data can be interrogated regarding the expression of the novel lincRNA as well as being able to investigate whether its expression correlates with that of nearby genes, towards a more generalized lncRNA expression hypothesis in the cancer under investigation. The controlled access data, such as TCGA data, may include two cohorts derived from two related cancers, where the novel lncRNA may be expressed. The many samples of the cohorts are used to interrogate the signal pattern of the novel lncRNA and the nearby genes towards further generalization of the relationship of this lncRNA and the nearby genes in two related cancer types.

After the raw data are retrieved and preprocessed as described in Materials and Methods, the processed file sets are placed within the storage structure that SeqCVIBE recognizes, and the database is updated. The three sets are ready for exploration and visualization rounds. In order to visualize the potential co-expression as well as its direction (positive or negative correlation) using the lab’s data, the user creates a dataset instance, excluding potentially poor-quality samples after inspection of the integrated interactive report. After instance creation, the user may generate a list of the nearby targets to produce average signal coverage plots in the respective SeqCVIBE section. In addition, the user can enter the novel lincRNA (approximate) co-ordinates and store them for further use in other analyses as well. Then, with the respective control buttons, the coverage of the requested samples is averaged and the respective figures are created in a grid format for all requested targets. Using the same gene and co-ordinate lists, the user may also request the calculation of actual expression values in various scales (e.g., RPKM, on-the-fly for the lincRNA) and perform pairwise correlation analysis with the list of transcripts and also hierarchical clustering. On a more genome-wide perspective, the user may also perform basic DGEA with the novel lincRNA attached to the rest of the genome annotation and interactively inspect the results. The user may then perform PCA analysis to investigate the ranking of the lincRNA concerning how much it contributes to the variance between knock-out and control samples when DGEA is also considered.

Similar processes can be performed with the other two datasets, resulting in a set of figures, expression values, and DGEA observations which, together, may generalize the novel lincRNA expression hypothesis and lead to further validation experiments and the design of new ones, potentially taking into account the response of the lincRNA to the drug administration in the public dataset. In particular, with large cohort datasets, the user may also visually inspect samples in the integrated genome browser and make sure that only samples of good quality are included in further interrogation. Finally, using the clear dataset instances, the user may save sessions and continue further exploration work at later time points, looking in more detailed DGEA and gene pairwise correlation outcomes in order to discover more potential interactors with the novel lincRNA under investigation.

*Usage scenario 2: My group studies liver development using the mouse as a model organism. I believe I have discovered a gene signature which significantly contributes in combination with the activity of known Transcription Factors (TFs) and I want to perform DGEA and clustering and correlation analysis. In addition, I want to check my gene signature on a mouse dataset studying brain development to check for potential correlations or conservation patterns.*

This scenario involves time-course RNA-Seq data generated and owned by the lab that has experimental evidence on this new gene signature contributing to liver development in the mouse. It also includes public RNA-Seq data retrieved from public repositories with no controlled access, such as the GEO. The lab data may be derived from young mice and at time points spanning several embryonic stages as well as newborn, intermediate, and adult mice. The public data may be derived from a similar experiment with the mouse as the experimental organism, but with possibly slightly differing time points and with the organ under developmental monitoring being the brain. The purpose of the second dataset is to examine whether the whole or part of the novel gene signature also participates in brain developmental pathways. In addition, the group wants to examine what the expression pattern is of the known TFs with respect to the brain dataset.

After raw data preprocessing and proper placing within the storage structure of SeqCVIBE, the two sets are ready for analysis and visualization. In order to visualize the potential co-expression of the TFs and whole or part of the gene signature and check whether there are correlation patterns at the same or prior time points, the user creates a dataset instance using the lab’s data, excluding potentially poor-quality samples after inspection of the integrated interactive report. After the instance creation, the user may create a list of the gene signature and the TFs to create average signal coverage plots in the respective SeqCVIBE section with the respective control buttons. The coverage of the requested target genes is averaged between samples and the respective figures are created in grid format for all requested genes. The user can inspect and verify co-expression patterns with the goal of locating gene signature members potentially affected by TF activity. Using the same gene and co-ordinate lists, the user may also request the calculation of actual expression values in various scales and perform pairwise correlation analysis with the list of transcripts and also between TFs and members of the gene signature. From a more genome-wide perspective, the user may also perform basic DGEA and interactively inspect the results. The user may then perform PCA across several time points to examine the ranking of the TFs with respect to how much they contribute to the variance between time points.

A comparable procedure can be followed with the brain dataset, resulting in a set of figures that can be compared and observations extracted regarding the behavior of the gene signature members in brain development. The TFs may also be examined for a potential role in this process through signal visualizations and correlation analyses. Basic DGEA in the brain dataset may also reveal additional targets that could complement the liver signature and create generalized results for both organs. Finally, PCA analysis in the brain dataset might reveal a different ranking for the importance of the TFs (if any) in the brain dataset. All the aforementioned procedures would be expected to lead to new observations, design of validation experiments, and formulation of additional hypotheses.

*Usage scenario 3: I teach Bioinformatics to Biology students and I want to prepare a workshop where I will demonstrate the basics of gene expression analysis using various examples of RNA-Seq data, as well as other generic aspects, such as genome browsing. I want a tool that can meet the requirements and also provide real-time analytics for discussion. Finally, as the workshop is addressed to bench biologists, I want to minimize the required computational skills.*

This scenario involves simple RNA-Seq datasets retrieved exclusively from public repositories with no controlled access, such as the GEO. The tutor will use two datasets of simple experimental designs from two different organisms. The first dataset is derived from human cancerous cell lines. The goal of the experiment is to test the efficacy of two dosages of a new drug candidate and see if a greater dose induces further response regarding cell line survival. The workshop attendees will inspect basic QC metrics, perform basic DGEA, and inspect the average signal of a few differentially expressed genes. They will also perform hierarchical clustering involving all three biological conditions (cancer cells without drug administration and two drug dosages) to visualize the different expression levels of differentially expressed genes. The second dataset comes from mice and concerns the comparison of wild-type adult mice against a model engineered to not express a particular gene affecting cognitive functions. The workshop attendees will again inspect basic QC metrics and perform basic DGEA. They will also visualize the signal of a gene cluster hypothetically affected by the knock-out of the aforementioned gene in order to check whether any other cluster members are affected by the knock-out. For both datasets, the attendees will explore genome browsing.

After raw data preprocessing and placing within the SeqCVIBE structure, the two sets are ready for analysis. For the first dataset (human cancerous cell lines), the QC reports reveal that one of the triplicate samples for the first dosage needs to be removed from further analysis. The attendees then proceed with the creation of a sub-dataset instance without the problematic replicate and continue with basic DGEA in pairwise mode for the comparisons of dosage 1 against no administration and dosage 2 against no administration. When DGEA is complete, the attendees use the interactive MA plot to select top up- and down-regulated genes. With these, they create a list and proceed to average signal visualization in the respective SeqCVIBE sections. After that, they move to hierarchical clustering, where they create a heatmap with Euclidean distance using the results of the DGEA (available directly in the respective section). The outcomes of both DGEA and clustering analysis reveal that the second dosage (greater) induces greater changes in the majority of the genes affected by the first and has effects on additional genes, as revealed by the greater numbers of differentially expressed genes. The attendees finally inspect the signal of individual samples in the genome browser of SeqCVIBE for certain genes of interest and also verify the poor quality of the sample that was excluded in terms of RNA signal as depicted in the genome browser.

For the second dataset (mouse), basic QC shows that all samples can be used for further analysis. However, MDS plots reveal that although the samples (triplicates) belonging to distinct biological conditions cluster well together, the dispersity of the samples across principal coordinates (the variability space) is greater, which is to be expected due to the in vivo nature of the experiment (animals instead of more controllable cell lines). The subsequent DGEA shows that a moderate number of genes is affected by the knock-out. The attendees then, as with the human dataset, browse the results of the DGEA through the interactive MA plot and table and experiment with the various filters available in SeqCVIBE’s DGEA section. The exploration reveals several genes which are affected by the knock-out, with some known to participate in cognitive function pathways, pointing to the existence of an affected gene network. Then, the attendees create a list of the genes in a gene cluster which is supposed to be affected by the knock-out. Using the RNA average signal functionality of SeqCVIBE, they visualize these genes both in a grid format with one figure for each individual gene, as well as a genome-browser instance-like format, where the whole genomic neighborhood is visualized. Finally, they inspect these genes per sample in the genome browser.

*Usage scenario 4: I am a Bioinformatics teaching assistant and I want to prepare a workshop addressed to math and/or computer science students without biological background. I will demonstrate the basics of gene expression analysis using various examples of RNA-Seq data but also explain biological signal and concepts such as exons. I want a tool that can meet the requirements, provide real-time analytics for discussion, and also provide intuitive visualizations for basic biological concepts regarding gene expression.*

Again, this education-based scenario involves simple RNA-Seq datasets retrieved from public repositories. The tutor will use two datasets of simple experimental designs from two different organisms. The first dataset is derived from a gene knock-out experiment on adult mice, involving three wild-type and three knock-out replicates. Since the course is addressed to attendees with little biological background, the tutor wishes to demonstrate the lack of expression and RNA signal for the gene being knocked out as well as display the altered expression of genes connected with the one knocked out. The tutor also wishes to introduce the attendees to the concept of differential expression. The second dataset is derived from the comparison of three human cell lines, one being normal while the other two are from two different cancer stages. The goal of the tutor with the second dataset is again to visualize the signal of known genes and also demonstrate clustering with respect to its biological significance and potential outcomes.

After raw data preprocessing and placing within the SeqCVIBE structure, the two sets are ready for analysis. The MultiQC report coupled with MDS analysis for the first dataset reveals that one replicate from the knock-out condition must be removed, therefore, the attendees proceed in creating a dataset instance without this replicate. Subsequently, they proceed to the signal viewer tabs of SeqCVIBE where they experiment with various visualizations for a gene set provided by the tutor. They experiment with the various views and distances from transcriptional start sites so as to understand the linear signal representations and understand exon coverage as well as genomic distances between possibly connected elements. Subsequently, they get introduced to DGEA using the respective functionality of SeqCVIBE, and they are asked to report top genes affected by the knock-out, after the tutor explains the concept of fold-change.

Regarding the second dataset, the QC shows that all samples can be used for analysis, therefore, a complete dataset instance is created. Similarly, as before, the attendees explore the signal of certain oncogenes and their expression according to tumor stage. Subsequently, they perform DGEA to identify genes potentially standing out in at least one of the three experimental conditions. Using the respective SeqCVIBE functionalities, they then create a further filtered list of differentially expressed genes which are then subjected to hierarchical clustering through the respective section of SeqCVIBE. The attendees are then requested to report differences between different clusters and identify patterns of co-expression across the three conditions.

### 4.2. Public Deployment Example of SeqCVIBE and Datasets Hosted

To demonstrate the functionalities and abilities of SeqCVIBE, a public instance was deployed within the framework of the Elixir-GR project (www.elixir-greece.org, accessed on 15 March 2022) and is available at http://elixir-seqcvibe.hybridstat.gr (accessed on 15 March 2022). Basic and free user authentication is required, also available through social login mechanisms. Detailed instructions regarding deployment as well as preprocessing are also available (see Materials and Methods). The RNA-Seq data hosted in the public instance of SeqCVIBE are derived from two major organisms, human and mouse, as they concern the majority of depositions in public repositories and are well studied. The raw data were retrieved from the GEO and ENA and span a variety of sample sizes and experimental conditions of general interest and impact. More specifically, we collected 29 (19 human, 10 mouse) datasets interrogating gene expression profiles across organ and tissue developmental stages and healthy and cancerous samples for specific cancer types as well as knock-out experiments with experimental controls, all from published studies. A detailed list of the dataset hosted in the public instance can be found in Supplemental File S1.

### 4.3. Investigation of the Role of WiNTRLINC1 in Wnt Signaling

**Figure 9:**
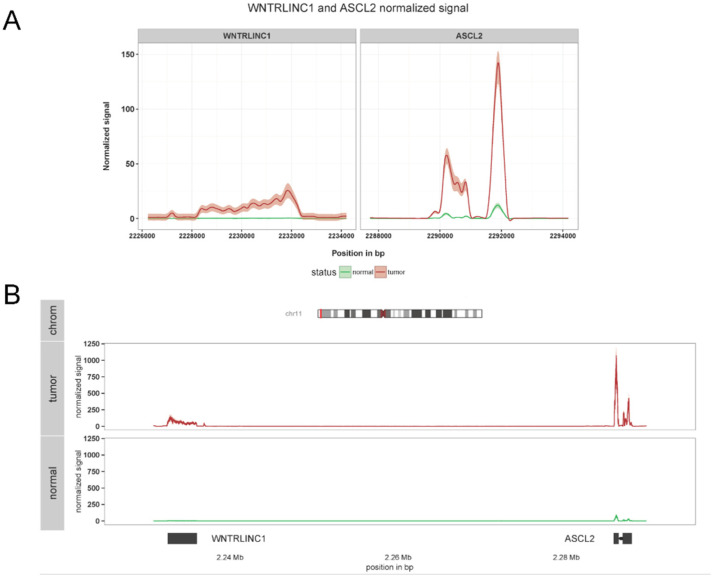
WiNTRLINC1 and ASCL2 co-expression in TCGA COAD data. SeqCVIBE was used to visualize the expression of the novel non-coding RNA WiNTRLINC1 as well as the ASCL2 gene, with which it forms a regulatory loop. The averaged signal visualization is derived from 434 tumor and 40 normal samples from the colon cancer dataset (COAD) from the TCGA network. (**A**) Distinct signal patterns of WiNTRLINC1 and ASCL2 in grid format. (**B**) The same signals visualized in the overall genomic neighborhood of WiNTRLINC1 and ASCL2. Structural information is also shown (bottom panel shows ASCL2 exons) while the top panel visualizes the chromosomal region where the particular genomic neighborhood is located.

The Wnt signaling pathway plays a major role in organ development by controlling important processes, such as stem cell differentiation and maintenance [20]. At the same time, Wnt pathway dysfunctions have been associated with various tumor types [21], especially in colorectal cancer [22]. We previously identified WiNTRLINC1, a nuclear long non-coding RNA which regulates the expression of its genomic neighbor ASCL2 towards the same direction (positive regulation) [23]. ASCL2 is a transcription factor that was previously shown to control the fate of intestinal stem cells as a target of the Wnt pathway [24]. Among other experimental and computational procedures, we sought to investigate whether the correlation of expression between WiNTRLINC1 and the nearby ASCL2 gene generalizes to human samples, outside the cell lines described in [23], using the whole COlorectal ADenocarcinoma (COAD) cohort of the TCGA project [25], containing many cancerous samples from which a subset had matched control healthy samples. After raw data acquisition and joint preprocessing, the data were imported to SeqCVIBE for further analysis. Subsequently, we computationally and visually (using the JBrowse instance in SeqCVIBE) identified samples of poor quality in both the control and disease groups and created a dataset instance in SeqCVIBE to narrow down our research. Among other analytics, we interrogated the dataset instance for both the expression and RNA-signal patterns of WiNTRLINC1 and ASCL2 in control and disease samples. This was performed by importing the non-generally (e.g., in Ensembl) annotated genomic co-ordinates of WiNTRLINC1 and also selecting the ASCL2 coordinates from the respective Ensembl annotation and subsequently calculating their read signal coverage on-the-fly. WiNTRLINC1 expression levels were also calculated on-the-fly using SeqCVIBE’s respective functionality. Our observations confirmed the hypothesis of the positive correlation between WiNTRLINC1 and ASCL2 in human colorectal samples and respective healthy samples (Figure 9).

## 5. Discussion

RNA-Seq constitutes the currently preferred technique for the quick and accurate investigation of gene expression profiles at the whole-genome level. As with data produced via most high-throughput techniques, the management and analysis of RNA-Seq data presents its own challenges. While the technique is essentially mature and so are most computational tools that address the analytical and visualization challenges posed by RNA-Seq data, there is a lack of integrated tools able to simultaneously provide a structured data management system as well as visualization and analytics capabilities. More specifically, while there are dedicated tools for data management and storage, quality control and basic signal analytics (e.g., genome browsers), DGEA, and additional expression analysis, such as clustering and correlation, the proper combination of all the above is rare, especially in a non-commercial setting. Whereas such combinations are achievable, they usually require extensive workflow authoring and gluing of many individual tools and components from different computational frameworks (e.g., different analytics languages), adding degrees of freedom to future maintenance and sustainability.

Three typical examples of lack of integration are certain types of Laboratory Information Management Systems (LIMS) which are able to accommodate sequencing experiment tracking and data management, DGEA tools, and packages focused solely on visualization. Regarding RNA-Seq LIMS, usually commercial and open-source (e.g., [26]), while they are mature platforms that excel in providing data management and experimental workflow execution tracking and management, they lack basic integration of RNA-Seq QC and analytics, other than the rudimentary reporting of numbers of sequenced and aligned reads as well as some basic statistics from base calling. Even though seemingly integrated solutions exist such as LabKey Server [27], this integration of RNA-Seq analytics does not ship out of the box and requires substantial additional programming and handling by experts or expensive consulting. While one could argue that analytics is not the main purpose of LIMS systems, this is not what most users believe, especially in the research and development domains, where analytics is part of routine data generation procedures. Although SeqCVIBE is not a LIMS, it manages to offer basic data management functionalities, coupled with powerful RNA-Seq analytics that may satisfy users seeking a basic integrated solution with many visual tools. Last but not least, we would like to stress again that LIMS systems are not designed for scientific analytics but mostly for tracking and auditing procedures, therefore, they cannot be directly compared with systems such as SeqCVIBE. However, our view is that they should be mentioned because latest versions of such systems (e.g., LabKey Server) seem to offer basic analytics and claim to integrate DGEA but not out of the box.

On the other hand, DGEA tools, which are mostly open-source, are also mature software components focusing almost solely on one, albeit very popular, aspect of RNA-Seq data, namely DGEA. They do not offer data management aside from tool-specific data structures required for the completion of the underlying analyses and they include only limited visualization tools, usually bound with DGEA visualization and diagnosis of issues related to it. Furthermore, they come with a variety of underlying statistical models, often leading to disconnected outcomes as a result of the different initial assumptions and requiring algorithms such as PANDORA [8] or the Harmonic mean p-value combination [28] to construct summarized results. SeqCVIBE comes not only with integrated basic DGEA and interactive visualization of the results but also a suite of DGEA-related analytics, such as clustering, PCA, and many types of correlation analyses. These features are effortlessly combined with the data management aspects outlined throughout our study.

Regarding the tools dedicated to visualization, these are most of the time either generic NGS signal visualization tools or even more general visualization tools for various data types. The first category includes genome browsers or tools that are able to summarize NGS signals over standard or custom regions [29,30]. The second category comprises tools for constructing generic analytics graphs (such as those with the ggplot2 R package), heatmaps, and other graphs. Neither category has many examples to present, with branches dedicated to RNA-Seq or out of the box integration with other RNA-Seq tools. SeqCVIBE includes average RNA-Seq signal visualization seamlessly integrated with DGEA and basic data management through interconnection of its various functionalities and minimal user effort. In addition, and to the best of our knowledge, SeqCVIBE is among the unique tools that offer real-time abundance estimation and visualization of non-annotated and possibly transcribed genomics regions, including, but not limited to, lincRNAs.

SeqCVIBE, apart from an RNA-Seq analytics tool, comprises also a database of pre-analyzed data, ready for exploration, querying, and additional on-the-fly analyses. While domain or disease-specific gene expression and multi-omics data and knowledge bases are appearing, making use of the wealth of public data [31,32,33,34], this knowledge usually is presented in tab-delimited and sometimes network format, with limited support for additional visualization tools, such as gene networks, expression heatmaps, scatterplots, and genome browsing support. SeqCVIBE can be used to create deeper knowledge instances for such use cases and domain-specific databases through the support of more extensive analytics, while at the same time offering the means to visualize RNA-Seq signals in graph and non-tabular formats. Furthermore, it could be used for on-the-fly DGEA, allowing users of such knowledge bases to pose direct questions to the gathered datasets, without relying only on the precalculated comparisons, and also to query non-annotated genomic regions of interest. Finally, it could be used for genome browsing on the domain-specific data, allowing the community to further contribute to a continuous quality assurance process, especially for disease-specific knowledge bases.

SeqCVIBE is currently focused on RNA-Seq signal and expression visualization, basic data management and offers a suite of classical expression analysis tools, integrated with its other functionalities, such as DGEA. Furthermore, although the required data preprocessing and application deployment process is well documented, some basic bioinformatics and server administration skills are still required. Our future plans include the complete automatization of the deployment process and CWL workflows for the data preprocessing. It also includes the addition of downstream expression analysis layers, such as ontological and biochemical pathway enrichment analysis and also gene set enrichment analysis through the integration of appropriate R and Bioconductor packages. Last but not least, we intend to add a gene network visualization to display interconnections within DGEA results, using appropriate databases, such as STRING.

## 6. Conclusions

Although RNA-Seq has prevailed as the major technique for the study of genome-wide gene expression profiles, the user communities are still struggling to create gold standards encompassing all related data analytics stages. While the data generation and analysis steps are well-defined and tools for each step are generally mature, there is a lack of integrated solutions covering most of them, from data management and genome browsing to biologically relevant outcomes. We have developed SeqCVIBE, a prototype solution to address the aforementioned issues in a biologist-friendly manner, while also providing implementation details and basic usage scenarios. A demo of SeqCVIBE has become accessible through the Elixir-GR project. In the future, we will extend the functionalities of SeqCVIBE with additional RNA-Seq-related analytics, such as biochemical pathway and gene set enrichment analysis, as well as gene network mapping.

## Data Availability

Not applicable.

## Supplementary Materials

The following supporting information can be downloaded at: https://www.mdpi.com/article/10.3390/mps5020027/s1, Supplemental File S1: Description of datasets included in the public deployment of SeqCVIBE, Supplemental File S2: Basic user guide of the application.
