# Supplementary material for: Interactive Analysis, Exploration, and Visualization of RNA-Seq Data with SeqCVIBE
Source: Methods Protoc. 2022 Mar 18;5(2):27. doi: 10.3390/mps5020027 (PMC8938808; doi:10.3390/mps5020027)
Supplement: Supplementary file 1 [file mps-05-00027-s001.zip › Supplemental File S1.pdf]

Interactive Analysis, Exploration and Visualization of RNA-Seq data with SeqCVIBE

Efthimios Bothos[1], Pantelis Hatzis[2], Panagiotis Moulos[2]

[1] Institute of Communications and Computer Systems, National Technical University of Athens, Athens, Greece.

[2] Institute for Fundamental Biomedical Research, Biomedical Sciences Research Center 'Alexander Fleming', Fleming 34, 16672, Vari, Greece.

Supplementary Material

Detailed description of the datasets hosted in the public deployment

To describe the datasets we use the parameter list that is given in Table 1. These parameters show the properties of the collected datasets.

Parameter	Description
**Accession ID**	Unique accession number of the dataset in the respective repository
**Platform - Protocol**	The sequencing platform used to produce the dataset
**Title**	The experiment title
**Short Summary**	A short description of the experiment
**Dataset URL**	A public link to the repository where raw data are maintained
**Experiment type**	Experiment type
**Experiment subject**	Experiment subject/goal
**Source**	Public repository source (GEO/ENA)
**# of Conditions**	Number of experimental conditions present in the experiment
**# of Samples**	Number of samples produced by the experiment
**Organism**	Model organism

Table S1: Parameters that describe the properties of the collected datasets.

The following Table 2 contains the detailed descriptions of the properties of the collected datasets based on the parameters mentioned above (Table 1).

<table>
<tr><td>Accession ID
GSE75711</td><td>Platform
Illumina HiSeq 2000</td><td colspan="2">Title
Generation of nephronal cells from human pluripotent stem cells via renal-vesicle formation</td></tr>
<tr><td colspan="2">Dataset URL
https://www.ncbi.nlm.nih.gov/geo/query/acc.cgi?acc=GSE75711</td><td>Experiment type
Organ Evolution</td><td>Experiment Subject
stem cells - nephronal cells generation</td></tr>
<tr><td>Conditions: 5</td><td>Samples: 15</td><td>Organism: Human</td><td>Source: GEO</td></tr>
<tr><td colspan="4">Short Summary: Human pluripotent stem cells (hPSC) provide a possible source for generation of kidney cells and organoids applicable in regenerative medicine, disease modeling and drug screening. By analyzing the effect of different factors on renal differentiation, we established an 8-day-protocol that steered hPSCs to the renal lineage by a step-wise process, segmented into mesoderm induction, intermediate mesoderm specification and metanephric induction outlining renal organogenesis. The differentiated</td></tr>
</table>

cells contain populations of SIX2+/CITED1+ metanephric mesenchyme- (MM) and of HOXB7+/GRHL2+ ureteric bud (UB)-like cells at the end of 6 days. Transcriptome analysis corroborated that the in vitro generated precursor cell types at day 8 resemble their renal vesicle counterparts in vivo. The cells were further differentiated in 2-dimensional culture into podocyte- and diverse tubular epithelial-like cell types, showing their nephrogenic potential. In 3-dimensional culture, the progenitors gave rise to renal vesicles, and upon mouse embryonic kidney re-aggregation they form tubular structures.			
Accession ID<br>GSE53667	Platform<br>Illumina HiSeq 2000	Title<br>Gene expression profiling in an induced pluripotent stem cell model of the developing human telencephalon: effect of heat shock and its potential impact on the development of neuropsychiatric disorders	
Dataset URL<br>https://www.ncbi.nlm.nih.gov/geo/query/acc.cgi?acc=GSE53667		Experiment type<br>Organ Evolution	Experiment Subject<br>Organ Evolution-neuropsychiatric disorders
Conditions: 2	Samples: 4	Organism: Human	Source: GEO
Short Summary: Schizophrenia (SZ) and autism spectrum disorders (ASD) are highly heritable neuropsychiatric/neurodevelopmental disorders, although environmental factors, such as maternal immune activation (MIA), play a role as well. Inflammatory cytokines appear to mediate the effects of MIA on neurogenesis and behavior in animal models. However, drugs and cytokines that trigger MIA can also induce a febrile reaction, which could have independent effects on neurogenesis through heat shock (HS)-regulated cellular stress pathways. However, this has not been well-studied. As a first step towards addressing the role of fever in MIA, we used a recently described model of human brain development in which induced pluripotent stem cells (iPSCs) differentiate into 3-dimensional neuronal aggregates that resemble a first trimester telencephalon. RNA-seq was carried out on aggregates that were heat shocked at 39oC for 24 hours, along with their control partners maintained at 37oC. Overall, 186 genes showed significant differences in expression following HS ($p<0.05$), including known HS-inducible genes, as expected, as well as those coding for NGFR and a number of SZ and ASD candidates, including SMARCA2, DPP10, ARNT2, AHI1 and ZNF804A. The degree to which the expression of these genes decrease or increase during HS is similar to that found in copy loss and copy gain CNVs, although the effects of HS are likely to be more transient.			
Accession ID<br>GSE56785	Platform<br>Illumina HiSeq 2000 - Illumina HiSeq 2500	Title<br>RNA-Seq characterization of human H1-derived NPC differentiation timecourse	
Dataset URL<br>https://www.ncbi.nlm.nih.gov/geo/query/acc.cgi?acc=GSE56785		Experiment type<br>Organ Evolution	Experiment Subject<br>Organ Evolution-neuronal precursor cells
Conditions: 7	Samples: 21	Organism: Human	Source: GEO
Short Summary: High resolution transcriptional profiling of H1-derived human neuronal precursor cells over a timecourse of differentiation in vitro.			
Accession ID<br>GSE69865	Platform<br>Illumina HiSeq 2000	Title<br>Role of Pou3f1 during mouse pluripotent stem cell neural fate commitment	
Dataset URL<br>https://www.ncbi.nlm.nih.gov/geo/query/acc.cgi?acc=GSE69865		Experiment type<br>Organ Evolution	Experiment Subject<br>stem cells
Conditions: 3	Samples: 6	Organism: Mouse	Source: GEO
Short Summary: The neural fate commitment of pluripotent stem cells requires repression of extrinsic inhibitory signals and activation of intrinsic positive transcription factors. However, it remains elusive how these two events are integrated to ensure appropriate neural conversion. Here, we show that Oct6 functions as an essential positive factor for neural differentiation of mouse embryonic stem cells (ESCs), specifically during the transition from epiblast stem cells (EpiSCs) to neural progenitor cells (NPCs). Chimera analysis showed that Oct6 knockdown leads to markedly decreased incorporation of ESC in neuroectoderm. By contrast, Oct6-overexpressing ESC derivatives preferentially contribute to neuroectoderm. Genome-wide ChIP-seq and RNA-seq analyses indicate that Oct6 is an upstream activator of neural lineage genes, and also a repressor of BMP and Wnt signalings. Our results establish Oct6 as a critical regulator that promotes neural commitment of pluripotent stem cells through a dual role: activating internal neural induction programs and antagonizing extrinsic neural inhibitory signals.			
Accession ID<br>GSE31271	Platform<br>Illumina Genome Analyzer IIx	Title<br>Control of Embryonic Stem Cell Lineage Commitment by Core Promoter Factor, TAF3	
Dataset URL<br>https://www.ncbi.nlm.nih.gov/geo/query/acc.cgi?acc=GSE31271		Experiment type<br>WT vs KO	Experiment Subject<br>stem cells
Conditions: 8	Samples: 16	Organism: Mouse	Source: GEO
Short Summary: We report that TAF3, a TBP-associated core promoter factor, is highly enriched in ES cells. In addition to its role in the core promoter recognition complex TFIID, genome-wide binding studies reveal that TAF3 localizes to chromosomal regions bound by CTCF and cohesin. Enrichment for TAF3/CTCF/cohesin bound regions distinguishes TAF3-activated from TAF3-repressed genes. Our findings support a new role of TAF3 in mediating long-range chromatin regulatory interactions to safeguard the finely-balanced transcriptional programs that give rise to pluripotency.			

<table>
<tr><td>Accession ID
PRJEB13935</td><td>Platform
Illumina HiSeq 2500</td><td colspan="2">Title
RNA-seq analysis of two cell line models of lung diseases in Project 3 of Open Targets - Epigenomes of Cell Lines</td></tr>
<tr><td colspan="2">Dataset URL
https://www.ebi.ac.uk/ena/data/view/PRJEB13935</td><td>Experiment type
Gene Expression Signature-Profiling</td><td>Experiment Subject
lung diseases</td></tr>
<tr><td>Conditions: 2</td><td>Samples: 4</td><td>Organism: Human</td><td>Source: ENA</td></tr>
<tr><td colspan="4">Short Summary: This experiment captures the baseline expression of two cell lines (A549, BEAS-2B) selected for Project 3, Open Targets (http://opentargets.org/, formerly CTTV). These cell lines are both possible models for lung disease. This RNA-seq experiment is being carried out as part of the Open Targets project to identify a gene expression signature of common immortalised cell lines/models. This signature will be used in combination with data from ChIP-seq experiments from the same cell lines against primary cells and tissues. The overall aim of the Open Targets Cell Line Epigenome project is to establish a systematic approach for the determination of human biological and disease relevance through the generation of transcriptomic and epigenomic data in cell lines of interest. Comparison of cell line mRNA expression and epigenome data with existing and newly generated reference data from human tissue and cell types will identify assay systems that will provide greater confidence in translating target biology and compound pharmacology to patients.</td></tr>
<tr><td>Accession ID
GSE51005</td><td>Platform
Illumina HiSeq 2000</td><td colspan="2">Title
Next generation sequencing of advanced non-castrate prostate cancer treated with docetaxel chemotherapy</td></tr>
<tr><td colspan="2">Dataset URL
https://www.ncbi.nlm.nih.gov/geo/query/acc.cgi?acc=GSE51005</td><td>Experiment type
Gene Expression Signature-Profiling</td><td>Experiment Subject
prostate cancer - drug response</td></tr>
<tr><td>Conditions: 2</td><td>Samples: 12</td><td>Organism: Human</td><td>Source: GEO</td></tr>
<tr><td colspan="4">Short Summary: Early chemotherapy for advanced/metastatic non-castration resistant prostate cancer (PCa) may improve overall patient survival. We studied the safety, tolerability and early efficacy of up-front docetaxel chemotherapy and androgen deprivation therapy (ADT) versus ADT alone for patients with newly-diagnosed advanced/metastatic PCa. As proof of concept, we undertook in vivo gene expression profiling by next generation RNA sequencing (RNA-Seq)</td></tr>
<tr><td>Accession ID
GSE70981</td><td>Platform
Illumina HiSeq 2500</td><td colspan="2">Title
Blimp1 controls plasma cell physiology and function</td></tr>
<tr><td colspan="2">Dataset URL
https://www.ncbi.nlm.nih.gov/geo/query/acc.cgi?acc=GSE70981</td><td>Experiment type
WT vs KO</td><td>Experiment Subject
Antibody-secreting plasma cells</td></tr>
<tr><td>Conditions: 3</td><td>Samples: 8</td><td>Organism: Mouse</td><td>Source: GEO</td></tr>
<tr><td colspan="4">Short Summary: Antibody-secreting plasma cells are the terminal stage of the B-cell lineage. Plasma cell differentiation requires a major resetting of gene expression to silence the B cell transcriptional program, whilst establishing secretory function and long-term survival. The transcription factors Blimp1 and Irf4 are essential for the initial differentiation of activated B cells to antibody-secreting cells, however their function in mature plasma cells remains elusive. We have found that while Irf4 was essential for plasma cell survival, Blimp1 was dispensable. Blimp1-deficient cells retained the unique plasma cell transcriptional signature, but lost the ability to secrete antibody or to maintain the characteristic size and ultrastructure of plasma cells. Blimp1 was required for full expression of many components of the unfolded protein response (UPR), including Xbp1 and Atf6, as well as for the appropriate processing of Igh mRNA. The overlap of Blimp1 and Xbp1 function was restricted to the UPR genes, with Blimp1 uniquely regulating activity of the mTOR pathway, plasma cell size and morphology. These studies establish Blimp1 as a major regulator of the UPR pathway that is also required for the unique metabolic requirements of plasma cells enabling the secretion of protective antibody.</td></tr>
<tr><td>Accession ID
GSE49555</td><td>Platform
Ion Torrent Proton</td><td colspan="2">Title
PR-SET7 inactivation causes hepatocyte necrosis and spontaneous development of hepatocellular carcinoma derived from ductal progenitor cells</td></tr>
<tr><td colspan="2">Dataset URL
https://www.ncbi.nlm.nih.gov/geo/query/acc.cgi?acc=GSE49555</td><td>Experiment type
WT vs KO</td><td>Experiment Subject
hepatocellular carcinoma</td></tr>
<tr><td>Conditions: 3</td><td>Samples: 8</td><td>Organism: Mouse</td><td>Source: GEO</td></tr>
<tr><td colspan="4">Short Summary: PR-SET7-mediated histone-4 lysine-20 methylation has been implicated in mitotic condensation, DNA damage response and replication licencing. Here we show that PR-SET7 function in the liver is pivotal for maintaining genome integrity. Hepatocyte-specific deletion of PR-SET7 in mouse embryos resulted in G2 arrest followed by massive cell death and defect in liver organogenesis. Inactivation at postnatal stages caused cell duplication-dependent hepatocyte necrosis with unusual features of autophagy, termed "endonucleosis". Necrotic death was accompanied by inflammation, fibrosis and compensatory growth induction of neighboring hepatocytes and resident ductal progenitor cells. Prolonged necrotic-regenerative cycles coupled with oncogenic STAT3 activation replaced pre-existing hepatocytes with hepatocellular carcinoma derived entirely from ductal progenitor cells. Hepatocellular carcinoma in these mice displays a cancer stem cell gene signature specified by the co-expression of ductal progenitor markers and oncofetal genes.</td></tr>
</table>

Accession ID<br>PRJEB65039	Platform<br>Illumina HiSeq 1500	Title<br>RNA-seq of gene expression changes in HEK293 cells after deletion of PRC1.6 complex component MGA	
Dataset URL<br>https://www.ebi.ac.uk/ena/data/view/PRJEB65039		Experiment type<br>WT vs KO	Experiment Subject<br>cancer
Conditions: 3	Samples: 6	Organism: Human	Source: ENA
Short Summary: MGA was depleted via Crisper/Cas9 in HEK293 cells and the resulting gene expression changes were profiled.			
Accession ID<br>PRJEB21725	Platform<br>NextSeq 500	Title<br>RNA-seq of coding RNA from DDX6 knockout human haploid (HAP1) cells	
Dataset URL<br>https://www.ebi.ac.uk/ena/data/view/PRJEB21725		Experiment type<br>WT vs KO	Experiment Subject<br>cancer
Conditions: 3	Samples: 8	Organism: Human	Source: ENA
Short Summary: We performed a genome-scale screen for suppressors of interferon stimulated gene (ISG) expression in human haploid cells (HAP1). DEAD-box helicase 6 (DDX6) was a significant hit. In order to validate DDX6 as a regulator of ISG expression, we created knockouts of DDX6 in HAP1 cellsusing CRISPR-Cas9 and performed RNA-seq on coding RNA from DDX6 KO and WT cells. This data was used to determine if ISGs were upregulated in DDX6 KO HAP1 cells.			
Accession ID<br>GSE78271	Platform<br>Illumina HiSeq 2500	Title<br>RNA-SEQ assay for wild type and CRISPR induced endoglin knockout human pulmonary artery smooth muscle cells (PASMC) cells	
Dataset URL<br>https://www.ncbi.nlm.nih.gov/geo/query/acc.cgi?acc=GSE78271		Experiment type<br>WT vs KO	Experiment Subject<br>pulmonary artery smooth muscle cells
Conditions: 2	Samples: 6	Organism: Human	Source: ENA
Short Summary: The goal of this study is to investigate the differential transcripted genes affected by CRISPR induced endoglin knockout in PASMC cells.			
Accession ID<br>GSE63485	Platform<br>Illumina HiSeq 2000	Title<br>RNA expression analysis upon JMJD1C depletion	
Dataset URL<br>https://www.ncbi.nlm.nih.gov/geo/query/acc.cgi?acc=GSE63485		Experiment type<br>WT vs KO	Experiment Subject<br>leukemia (AML)
Conditions: 3	Samples: 6	Organism: Human	Source: GEO
Short Summary: The AML1-ETO fusion protein, a transcription factor generated by the t(8;21) translocation in acute myeloid leukaemia (AML), dictates a leukemic program by increasing self-renewal and inhibiting differentiation. Here we demonstrate that the histone demethylase JMJD1C functions as a co-activator for AML1-ETO and is required for its transcriptional program. JMJD1C is directly recruited by AML1-ETO to its target genes and regulates their expression by maintaining low H3K9me2 levels. Analyses in JMJD1C knockout mice also establish a JMJD1C requirement for AML1-ETO's ability to increase proliferation. We also show a critical role for JMJD1C in the survival of multiple human AML cell lines, suggesting that it is required for leukemic programs in different AML cell types through its association with key transcription factors.			
Accession ID<br>GSE56691	Platform<br>Illumina HiSeq 2000	Title<br>Characterizing the contrasting roles of JMJD3 and UTX histone demethylases in T cell acute lymphoblastic leukemia	
Dataset URL<br>https://www.ncbi.nlm.nih.gov/geo/query/acc.cgi?acc=GSE56691		Experiment type<br>WT vs KO (Time Points)	Experiment Subject<br>leukemia (T-ALL)
Conditions: 4	Samples: 12	Organism: Human	Source: GEO
Short Summary: T-cell acute lymphoblastic leukemia (T-ALL) is an immature hematopoietic malignancy driven mainly by oncogenic activation of NOTCH1 signaling. In this study we chemically inhibited the H3K27me3 demethylase JMJD3 using the GSKJ4 inhibitor and assayed for genome-wide changes in H3K27me3 and JMJD3 enrichment. This piece of data was further integrated to expression changes using RNA sequencing as well as ChIP-Sequencing analysis of H3K27me3 upon genomic knock-down of JMJD3 and UTX. These results, coupled to genomic analysis of primary samples for the genomic status of the UTX gene in T-ALL, helped us to identify a hitherto unknown role of JMJD3 as an oncogenice facilitator in leukemia whereas UTX seems to play a tumor suppressor role.			
Accession ID	Platform	Title	

<table>
<tr><td>PRJEB27811</td><td>Illumina HiSeq 3000</td><td colspan="2">RNA-Seq of human fetal hearts at 9, 12 and 16 weeks of gestation</td></tr>
<tr><td colspan="2">Dataset URL
https://www.ebi.ac.uk/ena/data/view/PRJEB27811</td><td>Experiment type
Organ Evolution</td><td>Experiment Subject
Organ Evolution-human heart</td></tr>
<tr><td>Conditions: 3</td><td>Samples: 3</td><td>Organism: Human</td><td>Source: ENA</td></tr>
<tr><td colspan="4">Short Summary: RNA-Seq of human fetal hearts at 9, 12 and 16 weeks of gestation</td></tr>
<tr><td>Accession ID
GSE72536</td><td>Platform
Illumina HiSeq 2000</td><td colspan="2">Title
Gene expression analysis of TIL rich HPV driven head and neck tumours reveals a distinct B-cell signature when compared to HPV independent tumours</td></tr>
<tr><td colspan="2">Dataset URL
https://www.ncbi.nlm.nih.gov/geo/query/acc.cgi?acc=GSE72536</td><td>Experiment type
Gene Expression Signature-Profiling</td><td>Experiment Subject
HPV - Head and Neck cancer</td></tr>
<tr><td>Conditions: 1</td><td>Samples: 23</td><td>Organism: Human</td><td>Source: GEO</td></tr>
<tr><td colspan="4">Short Summary: Human papilloma virus (HPV) associated head and neck squamous cell carcinoma (HNSCC) has a better prognosis than HPV(-) negative cancer. This may be due, in part, to the higher number of tumour infiltrating lymphocytes (TIL) in HPV(+) tumours. We used RNAseq to evaluate whether these differences in clinical behaviour could be explained simply by a numerical difference in TILs or whether there was a fundamental difference between TILs in these two settings.</td></tr>
<tr><td>Accession ID
GSE62642</td><td>Platform
Illumina HiSeq 2500</td><td colspan="2">Title
RNA-Seq Analysis in purified iPS cell-derived neuronal samples</td></tr>
<tr><td colspan="2">Dataset URL
https://www.ncbi.nlm.nih.gov/geo/query/acc.cgi?acc=GSE62642</td><td>Experiment type
Gene Expression Signature-Profiling</td><td>Experiment Subject
Parkinson's disease</td></tr>
<tr><td>Conditions: 7</td><td>Samples: 14</td><td>Organism: Human</td><td>Source: GEO</td></tr>
<tr><td colspan="4">Short Summary: We characterized the gene expression differences in mDA neurons from all PD (Parkinson's disease) cases (6 independent samples) and controls (8 independent samples), identifying 1,028 differentially expressed genes making up the PD expression signature. Strikingly, MAOB gene was identified as significantly differentially expressed (p = 0.046). The heat map clearly differentiates cases from controls, where interestingly most differentially expressed genes had lower expression in PD cases compared to controls. In the clustering, the RNA expression pattern of the control (C2) with a family history of PD located close to the PD expression signature suggested a susceptibility to PD.</td></tr>
<tr><td>Accession ID
GSE58326</td><td>Platform
Illumina HiSeq 2000</td><td colspan="2">Title
Global analysis of ZNF217 chromatin occupancy in the breast cancer cell genome reveals an association with Eralpha</td></tr>
<tr><td colspan="2">Dataset URL
https://www.ncbi.nlm.nih.gov/geo/query/acc.cgi?acc=GSE58326</td><td>Experiment type
Gene Expression Signature-Profiling</td><td>Experiment Subject
breast cancer</td></tr>
<tr><td>Conditions: 2</td><td>Samples: 6</td><td>Organism: Human</td><td>Source: GEO</td></tr>
<tr><td colspan="4">Short Summary: The ZNF217 gene, encoding a C2H2 zinc finger protein, is located at 20q13 and found amplified and overexpressed in greater than 20% of breast tumors. Current studies indicate ZNF217 drives tumorigenesis, yet the regulatory mechanisms of ZNF217 are largely unknown. Because ZNF217 associates with chromatin modifying enzymes, we postulate that ZNF217 functions to regulate specific gene signaling networks. Here, we present a large-scale functional genomic analysis of ZNF217, which provides insights into the regulatory role of ZNF217 in MCF7 breast cancer cells. Results: ChIP-seq analysis reveals that the majority of ZNF217 binding sites are located at distal regulatory regions associated with the chromatin marks H3K27ac and H3K4me1. Analysis of ChIPseq transcription factor binding sites shows clustering of ZNF217 with FOXA1, GATA3 and ERalpha binding sites, supported by the enrichment of corresponding motifs for the ERalpha-associated cisregulatory sequences. ERalpha expression highly correlates with ZNF217 in lysates from breast tumors (n=15), and ERalpha co-precipitates ZNF217 and its binding partner CtBP2 from nuclear extracts. Transcriptome profiling following ZNF217 depletion identifies differentially expressed genes co-bound by ZNF217 and ERalpha; gene ontology suggests a role for ZNF217-ERalpha in expression programs associated with ER+ breast cancer studies found in the Molecular Signature Database. Data-mining of expression data from breast cancer patients correlates ZNF217 with reduced overall survival in multiple subtypes. Conclusions: Our genome-wide ZNF217 data suggests a functional role for ZNF217 at ERalpha target genes. Future studies will investigate whether ZNF217 expression contributes to aberrant ERalpha regulatory events in ER+ breast cancer and hormone resistance</td></tr>
<tr><td>Accession ID
GSE40131</td><td>Platform
Illumina Genome Analyzer IIx</td><td colspan="2">Title
Synthetic cationic peptide IDR-1018 modulates human macrophage differentiation</td></tr>
<tr><td colspan="2">Dataset URL
https://www.ncbi.nlm.nih.gov/geo/query/acc.cgi?acc=GSE40131</td><td>Experiment type
Gene Expression Signature-Profiling</td><td>Experiment Subject
immunology</td></tr>
<tr><td>Conditions: 2</td><td>Samples: 6</td><td>Organism: Human</td><td>Source: GEO</td></tr>
<tr><td colspan="4">Short Summary: Synthetic, innate defense regulators (IDR) peptides, designed based on natural host defenses peptides, have enhanced immunomodulatory activities and reduced toxicity leading to protection in infection and inflammation models that is dependent on macrophages/monocytes. Here we measured the effect of IDR-1018 on macrophage gene expression during differentiation.</td></tr>
</table>

Differentiation in the presence of IDR-1018 induced a unique signature of immune responses suggesting that IDR-1018 drives macrophage differentiation towards an intermediate M1-M2 state, enhancing anti-inflammatory functions while maintaining certain pro-inflammatory activities important to the resolution of infection.			
Accession ID<br>PRJEB16733	Platform<br>Illumina HiSeq 2500	Title<br>Expression profiling of 3 and 24 months mouse skeletal muscle	
Dataset URL<br>https://www.ebi.ac.uk/ena/data/view/PRJEB16733		Experiment type<br>Organ Evolution	Experiment Subject<br>Organ Evolution-skeletal muscle
Conditions: 2	Samples: 2	Organism: Mouse	Source: ENA
Short Summary: Aging in multicellular organisms is characterized by gradual decline of organ functionality. To study the aging process, we used mRNA sequencing (mRNA-seq) to identify gene expression changes during aging in healthy mice. Skeletal muscle tissues of wild-type mice at 3 months and 24 months of age were collected and mRNA-seq libraries were prepared and sequenced on a HiSeq2500 by single-end sequencing with 100 bp read length. Analysis of the expression profiles of aged skeletal muscle tissues showed decreased mRNA levels of genes function in lipid metabolism, peptidase activity and response to stimulus.			
Accession ID<br>GSE58283	Platform<br>Illumina HiSeq 2500	Title<br>Gene expression profiling of mouse bone marrow-derived macrophages	
Dataset URL<br>https://www.ncbi.nlm.nih.gov/geo/query/acc.cgi?acc=GSE58283		Experiment type<br>WT vs KO	Experiment Subject<br>immunology
Conditions: 2	Samples: 8	Organism: Mouse	Source: GEO
Short Summary: Bone marrow (BM) cells were obtained by flushing the long bones of 8-week old C57BL/6 mice. BM cells were then were plated in macrophage SFM medium (Life Technologies) supplemented with penicillin-streptomycin and CSF-1 (Peprotech, 100 ng/ml) and cultured for one week to allow macrophage differentiation. BMDMs were polarized by adding IL-4 to the medium (40 ng/ml, Peprotech) for 72h or left untreated.			
Accession ID<br>GSE59017	Platform<br>Illumina Genome Analyzer IIx	Title<br>Next Generation Sequencing Facilitates Quantitative Analysis of Wild Type and dKO round spermatids Transcriptomes	
Dataset URL<br>https://www.ncbi.nlm.nih.gov/geo/query/acc.cgi?acc=GSE59017		Experiment type<br>WT vs KO	Experiment Subject<br>transcriptome profiling
Conditions: 2	Samples: 2	Organism: Mouse	Source: GEO
Short Summary: Next-generation sequencing (NGS) has revolutionized systems-based analysis of cellular pathways. The goals of this study are to compare NGS-derived WT and dKO round spermatids transcriptome profiling (RNA-seq)			
Accession ID<br>GSE57397	Platform<br>Illumina HiSeq 2000	Title<br>Transriptional profiling upon heat shock and recovery in cells deficient for FBXW7 and their wild type counterpart.	
Dataset URL<br>https://www.ncbi.nlm.nih.gov/geo/query/acc.cgi?acc=GSE57397		Experiment type<br>WT vs KO	Experiment Subject<br>cancer - transcriptome profiling
Conditions: 4	Samples: 8	Organism: Mouse	Source: GEO
Short Summary: FBXW7 modulates stress response by post-translational modification of HSF1 HSF1 orchestrates the heat-shock response upon exposure to heat stress and activates a transcriptional program vital for cancer cells. Genes positively regulated by HSF1 show increeased expression during heat shock while their expression is reduced during recovery. Genes negatively regulated by HSF1 show the opposite pattern. In this study we utilized the HCT116 FBXW7 KO colon cell line and its wild type counterpart to monitor gene expression changes during heat shock (42oC, 1 hour) and recovery (37oC for 2 hours post heat shock) using RNA sequencing. These results revealed that the heat-shock response pathway is prolonged in cells deficient for FBXW7			
Accession ID<br>GSE79183	Platform<br>Illumina HiSeq 2500	Title<br>Changes in RNA expression in human oral cavity carcinoma cells as a result of LDB1 reduction	
Dataset URL<br>https://www.ncbi.nlm.nih.gov/geo/query/acc.cgi?acc=GSE79183		Experiment type<br>WT vs KO	Experiment Subject<br>oral cavity carcinoma
Conditions: 2	Samples: 6	Organism: Human	Source: GEO
Short Summary: The study was designed to identify differential expressed genes between human oral cavity carcinoma cell lines with and without LDBI knockout			
Accession ID	Platform	Title	

PRJEB27927	Illumina HiSeq 4000	RNA-seq experiment to investigate the effect of inactivating the Apoptosis Antagonizing Transcription Factor (Aatf)	
Dataset URL<br>https://www.ebi.ac.uk/ena/data/view/PRJEB27927		Experiment type<br>WT vs KO	Experiment Subject<br>Apoptosis Antagonizing Transcription
Conditions: 2	Samples: 8	Organism: Mouse	Source: ENA
Short Summary: Human nephronophthisis and related ciliopathies suggest a link between ciliary signaling defects and altered DNA damage responses. The goal of our study is to elucidate the molecular link of both signaling systems as well as the role of altered DNA damage responses in kidney degeneration and fibrosis. The kinase-regulated DNA damage response target Apoptosis Antagonizing Transcription Factor (Aatf) is a master regulator of the p53 response. Upon genetic deletion of Aatf in renal tubular cells we induce progressive renal failure and a phenotype closely resembling human nephronophthisis in mice and are able to show Aatf as a regulator of primary cilia and modulator of the DNA damage response connecting two pathogenetic concepts of nephronophthisi and nephronophthisi-related ciliopathies. The analysis of the RNA-sequencing of four Aatf-knockout mice and four wildtype mice supports the experimental findings.			
Accession ID<br>PRJEB32551	Platform<br>Illumina HiSeq 2500	Title<br>Timeline RNAseq of fibroblast to neuron direction conversion	
Dataset URL<br>https://www.ebi.ac.uk/ena/data/view/PRJEB32551		Experiment type<br>Organ Evolution	Experiment Subject<br>Neuron evolution
Conditions: 1	Samples: 7	Organism: Human	Source: ENA
Short Summary: Timeline RNAseq to identify gene expression dynamics over the course of conversion of fibroblasts into induced neurons.			
Accession ID<br>PRJEB31080	Platform<br>Illumina HiSeq 3000	Title<br>RNA-seq of human cardiovascular endothelial cell line HUVEC, treated with different media	
Dataset URL<br>https://www.ebi.ac.uk/ena/data/view/PRJEB31080		Experiment type<br>WT vs KO	Experiment Subject<br>Cardiovascular endothelial cells
Conditions: 2	Samples: 8	Organism: Human	Source: ENA
Short Summary: Human cardiovascular endothelial cells were treated with different media for seven days. Subsequently, total RNA was isolated and purified. After rRNA removal, the samples were sequenced using HiSeq3000 (Illumina).			
Accession ID<br>PRJEB32967	Platform<br>NextSeq 500	Title<br>RNA-seq comparison of fibro-adipogenic progenitors (FAPs) from wild type, mdx and cardiotoxin-injured mice	
Dataset URL<br>https://www.ebi.ac.uk/ena/data/view/PRJEB32967		Experiment type<br>WT vs KO	Experiment Subject<br>fibro-adipogenic progenitors
Conditions: 3	Samples: 12	Organism: Mouse	Source: ENA
Short Summary: We purified FAPs from the hind limbs of wild type, mdx and cardiotoxin-injured mice in order to unveil changes in their transcriptomes. RNA was isolated directly from sorted cells and analyzed by 3' RNA sequencing.			
Accession ID<br>PRJEB33005	Platform<br>NextSeq 501	Title<br>Transcriptome analysis of human brain microvascular endothelial cells response to Borrelia burgdorferi and its antigen Erp23 using RNA-seq	
Dataset URL<br>https://www.ebi.ac.uk/ena/data/view/PRJEB33005		Experiment type<br>Expression Signature-Profiling	Experiment Subject<br>Brain microvascular endothelial cells
Conditions: 3	Samples: 9	Organism: Human	Source: ENA
Short Summary: Using RNA sequencing to map differentially expressed genes in human brain microvascular endothelial cells challenged with Borrelia burgdorferi or its ligand Erp23.			

Table S2: Detailed descriptions of the collected datasets.
