# Supplementary material for: Interactive Analysis, Exploration, and Visualization of RNA-Seq Data with SeqCVIBE
Source: Methods Protoc. 2022 Mar 18;5(2):27. doi: 10.3390/mps5020027 (PMC8938808; doi:10.3390/mps5020027)
Supplement: Supplementary file 1 [file mps-05-00027-s001.zip › Supplemental File S2.pdf]

SeqCVIBE

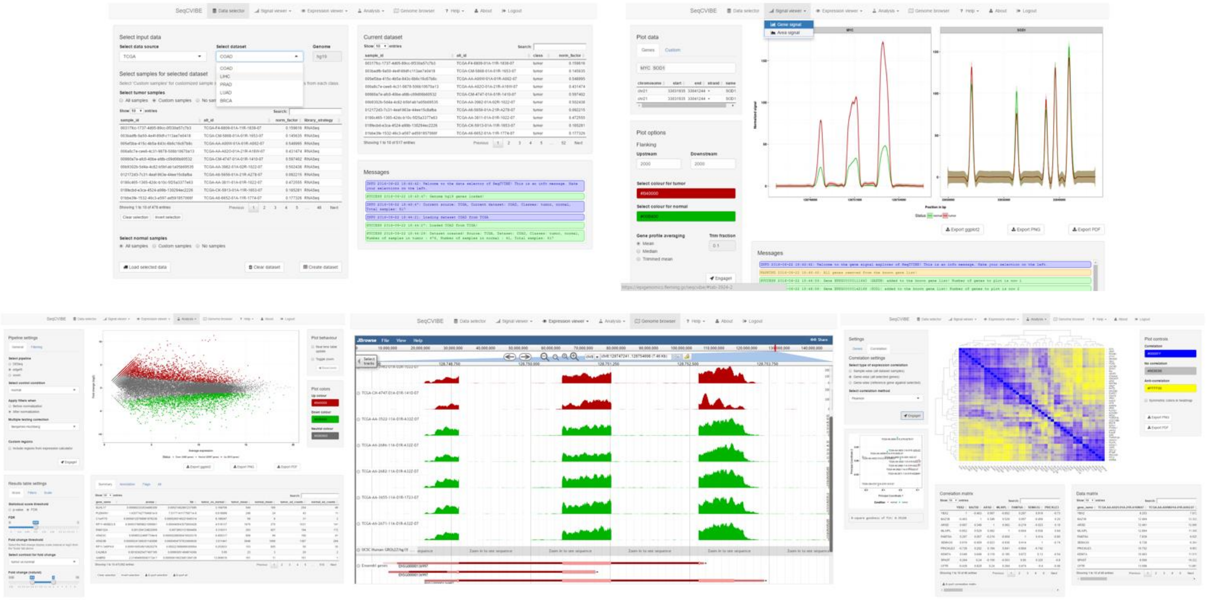

User Manual

Athens, 2022

Table of Contents

1. Overview 3
  1.1. Platform overview 3
  1.2. Data management 3
  1.3. Data selector 4
  1.4. Sessions 5
2. Signal viewer 6
  2.1. Gene signal 6
    2.1.1. Gene plotting 6
    2.1.2. Custom region plotting 7
  2.2. Area signal 8
    2.2.1. Custom genomic area 9
    2.2.2. Around gene area 9
  2.3. Plot options 9
  2.4. Exporting plots 10
3. Expression viewer 10
  3.1. Known genes expression 10
  3.2. Expression calculator 11
  3.3. Expression settings 12
  3.4. Exporting table 13
4. Analysis 13
  4.1. Differential Expression analysis 13
    4.1.1. Pipeline settings 14
    4.1.2. Results table 15
    4.1.3. Plot options 16
  4.2. Clustering analysis 16
    4.2.1. Gene settings 16
    4.2.2. Heatmap settings 17
  4.3. Correlation analysis 17
    4.3.1. Settings 17
    4.3.2. Additional matrices 18
  4.4. MDS/PCA Analysis 19
  4.5. Settings 19
  4.6. Coordinate plotting 20
5. Genome browser 21

1. Overview

Analyzing and visualizing an RNA-Seq analysis entirely is quite a demanding task. There is a wide range of tools required in the process of depicting all the significant information produced by an experiment. In almost all cases they have to be combined in a well-orchestrated way in order for a complete picture to be drawn and a full image of the analysis to be delivered, as there is no such thing as a 'swiss army knife' for this process.

1.1. Platform overview

The data exploration platform is an R/Shiny tool that aggregates all the analyses involved in an RNA-Seq experiment in a well-organized and clear manner. The user can select an analysis from a variety of experimental designs derived from two of the popular public dataset repositories, Gene Expression Omnibus (GEO) and European Nucleotide Archive (ENA). These experiments cover areas like profiling of genes involved in cancer or other neurological diseases, organ evolution, stem cell expression signature profiling etc and have been selected for the pilot deployment of the platform.

These analyses are fully configurable allowing the user to control parameters in very simple steps, especially compared to the often confusing way a command line tool works. In addition to that, all analyses are reproducible and can be easily saved and restored from specific time points.

1.2. Data management

The **Data management** tab comprises the starting point of the analysis. The user is prompted to select one of the available experiments to analyze. The selection process involves selecting a source (at present GEO, ENA but can be for example the name of a dataset provider) and a dataset from this source. After selection, the dataset is loaded and some basic information about the dataset are being displayed along with informative messages in case an error occurs.

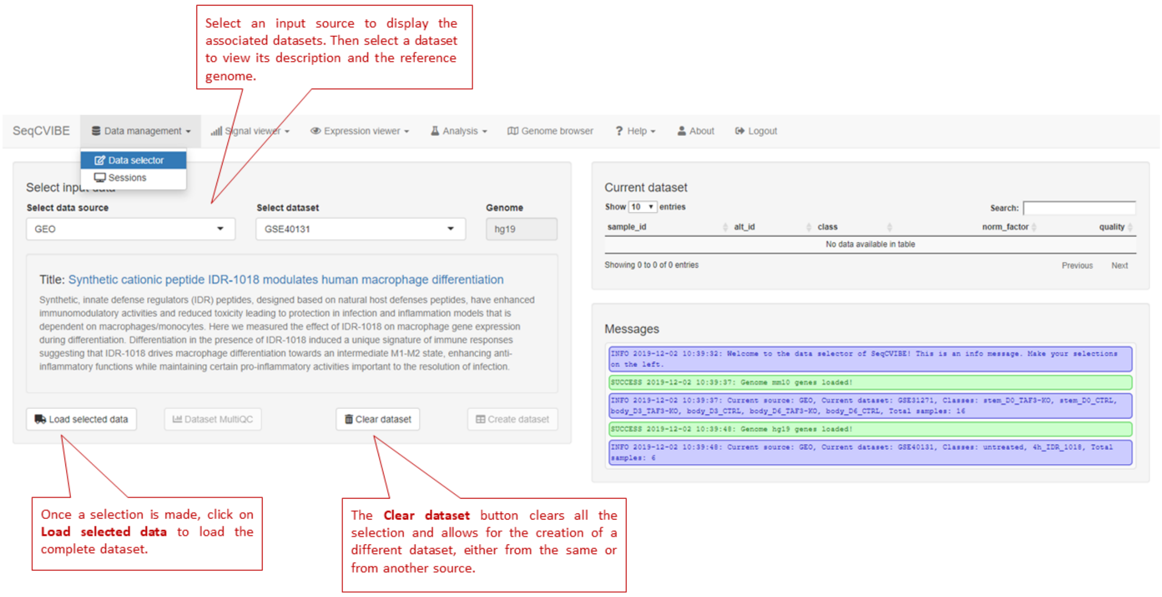

Figure S1: The data selector before a dataset is loaded.

1.3. Data selector

The **Data selector** sub-menu displays a panel which allows the user to select an input from one of the currently available sources. Each source is connected to the datasets derived from this source and each dataset is connected to a reference species genome which is always automatically selected based on the dataset.

Once the selection is made, the relevant short summary of the experiment will appear below. Clicking on the title will redirect the user to the relevant source page (currently the available ones are Gene Expression Omnibus (GEO) and European Nucleotide Archive (ENA)) were the analysis design and details are more thoroughly described.

The selected dataset is loaded by clicking the **Load selected data** button. Loading the dataset allows for exclusion of specific samples per condition or exclusion of all samples belonging to a single condition of the experiment. This way, the user has the ability to create a fully customizable (and recoverable) dataset.

After a dataset is loaded, the option to inspect its quality is enabled with MultiQC. By clicking on **Dataset MultiQC,** a new window presenting an aggregated analysis of all quality metrics for all samples of the dataset will appear. The quality metrics have been constructed using the FastQC package and viualized through the MultiQC package. This inspection can prove essential in the selection or exclusion of samples to be included in the final dataset based on specific properties the user might find significant.

When the dataset selections are made the user can hit the **Create dataset** button to finalize the dataset to be analyzed/explored. This will "lock" the sample options and all specific inclusions applied and will set the basis for all subsequent analyses. The included samples of the final dataset will be displayed in a table on the panel on the right named 'Current dataset'. After this point, anytime users wanting to run a new analysis with a new dataset they have to **Clear dataset** and start over again.

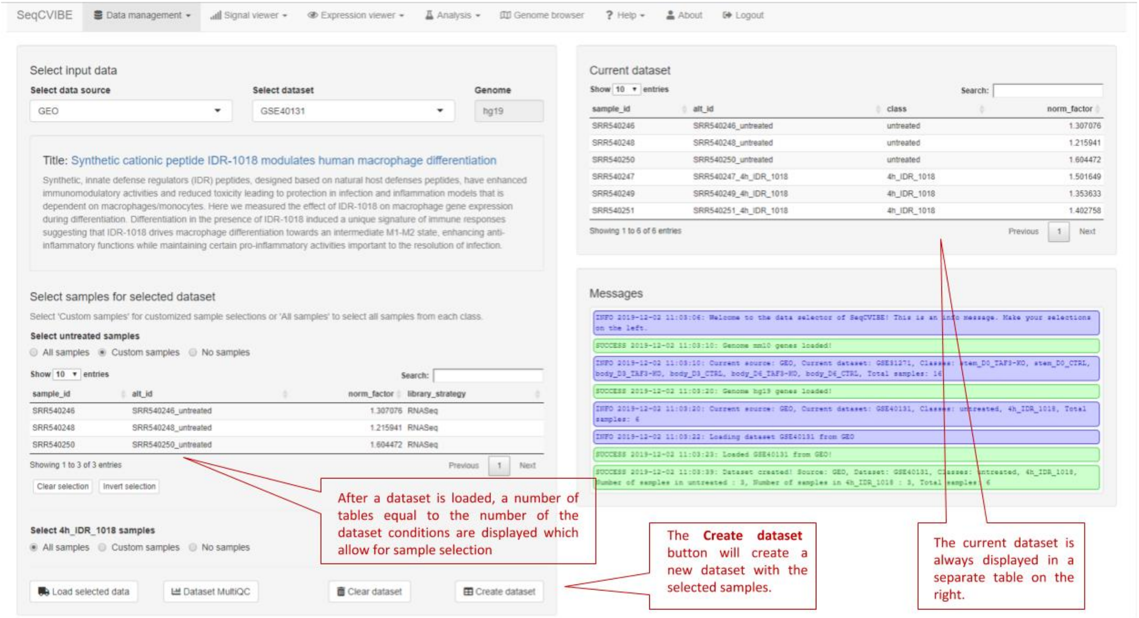

Figure S2: The data selector after a dataset is loaded.

1.4. Sessions

Bookmark's purpose is quite straight-forward. It places a bookmark in every tab and every visualization of the current status of SeqCVIBE, and allows for easy restore to that checkpoint at anytime. In other words it generates a save screenshot which can be loaded by any user of the group. At the end of the analysis the user has to enter a name for the bookmark and click the 'Add Bookmark' button. Then the bookmark will be added to the list of available bookmarks and any user will have the ability to restore it to its current state by clicking on the relevant URL. After the bookmarked state is fully loaded, users can make additional changes and save the analysis on top of the previous bookmark.

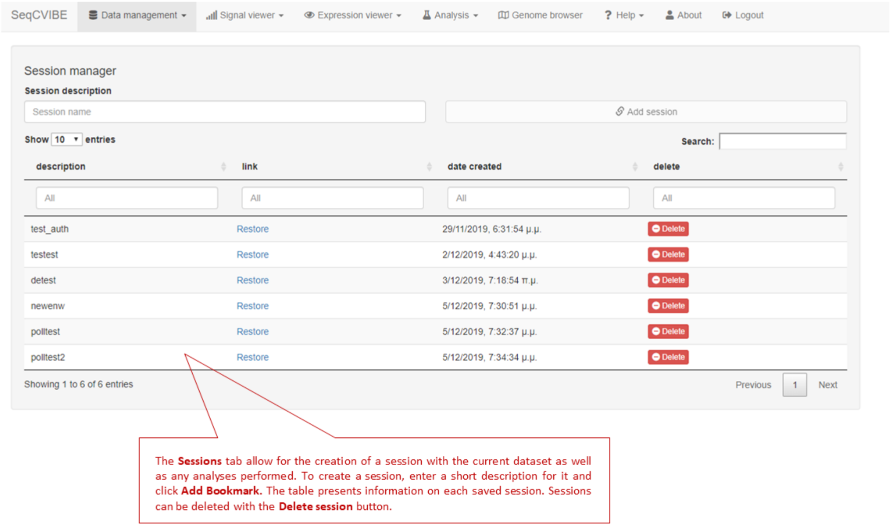

Figure S3: The session manager

2. Signal viewer

The Signal viewer allows for visual representation of one or more genomic regions of interest with reference to normalized signal. There are two options available, Gene Signal and Area Signal.

2.1. Gene signal

Gene signal plots the combined normalized signal of the selected gene(s) features/conditions. The user can select plotting input between specific genes by their name, or by a custom input genomic region. Furthermore, the user can optionally change the upstream and downstream regions to be plotted, select the different colours for each feature/condition of the experiment and finally, select one method for the gene profile averaging among Mean, Median and Trimmed mean. It the last case, the trimming fraction can also be provided.

By hitting the **Engage** button, the signal of all the selected genes and custom areas will be calculated and averaged. Once calculations are complete the plot will appear in the center of the tab. If there's more than one selection of gene or custom region (or both) to be displayed, an equivalent number of plots will be displayed in the same tab.

2.1.1. Gene plotting

The user can request for one or more genes to be plotted by clicking on the textbox below 'Plot data'. A drop-down menu of all known gene names of the concerning experiment species will appear. The user can search for genes by typing first letters. Alternatively, the user can always input the gene name considered, however this is not advised due to the fact that gene name is case sensitive. If the gene is

selected successfully it will be displayed as a new entry in the table below. There, its genomic coordinates will be also be displayed.

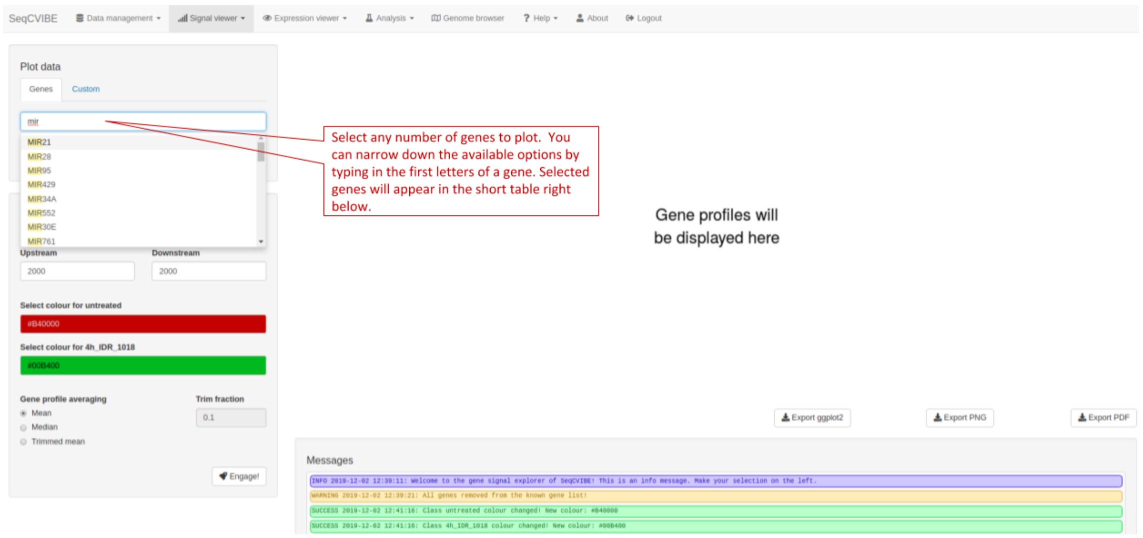

Figure S4: Selecting genes to create RNA signal profiles for.

2.1.2. Custom region plotting

Apart from plotting a specific gene region there is an option to plot the signal over custom genomic areas of interest, by selecting the 'Custom' tab in the 'Plot data' panel. The user will be prompted to fill in the requested area coordinates, like chromosome number, start-end position of the area and strand. The given area should also be given a unique name to be separated from any other regions that may be entered. The **Add** and **Remove** buttons below allow for the addition of a new region to the table of regions to be plotted or the removal of the selected ones.

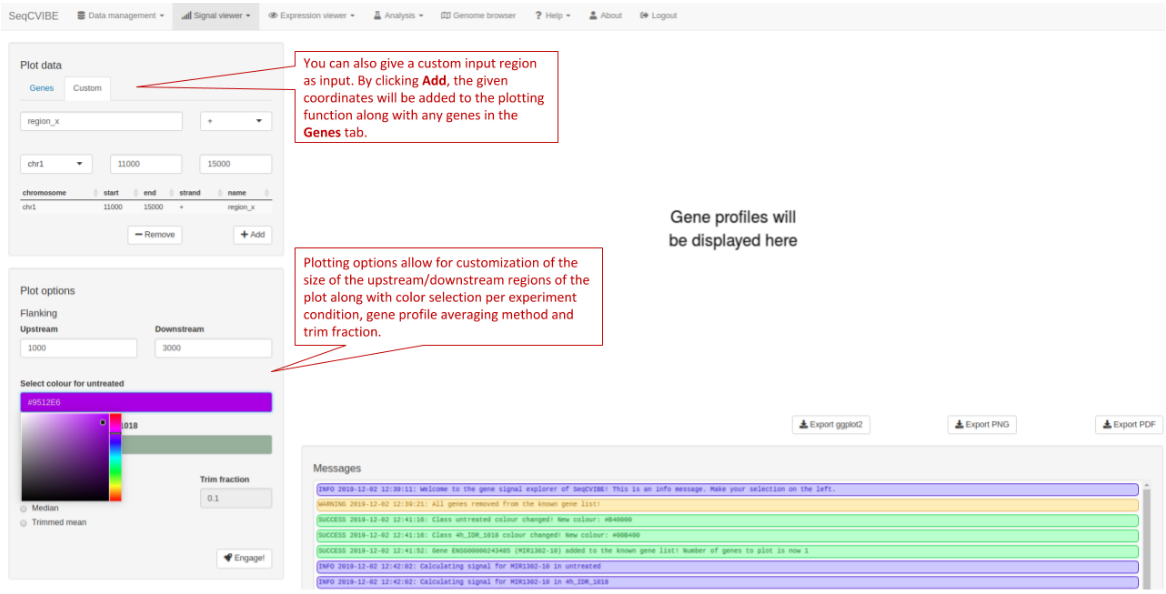

Figure S5: Defining customizing regions over which to calculate RNA signal and customizing plot colors.

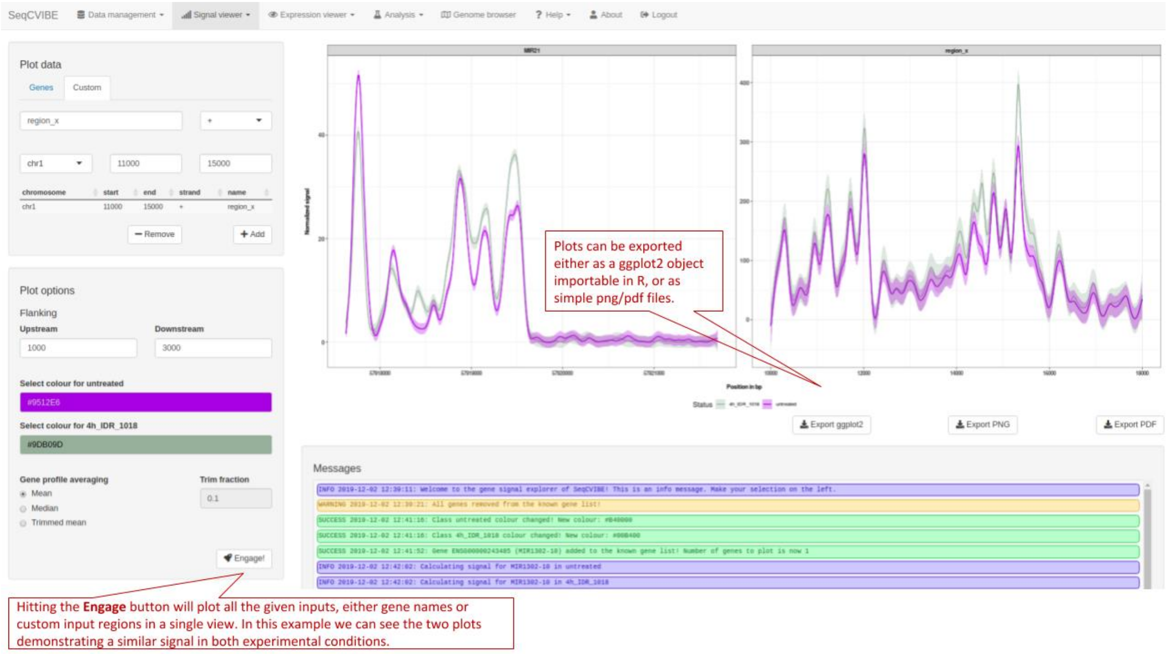

Figure S6: Creating RNA signals after choosing genomic regions and plot options.

2.2. Area signal

The Area signal works in a similar manner to the Gene signal tool. Its difference lies in the display style of the results which is of a wider area than what's just covered by the gene and resembles that of a genome browser. It can also plot multiple genes in a region without separating the plots for every region, like it happens with the Gene signal option, as long as they are in the same chromosome.

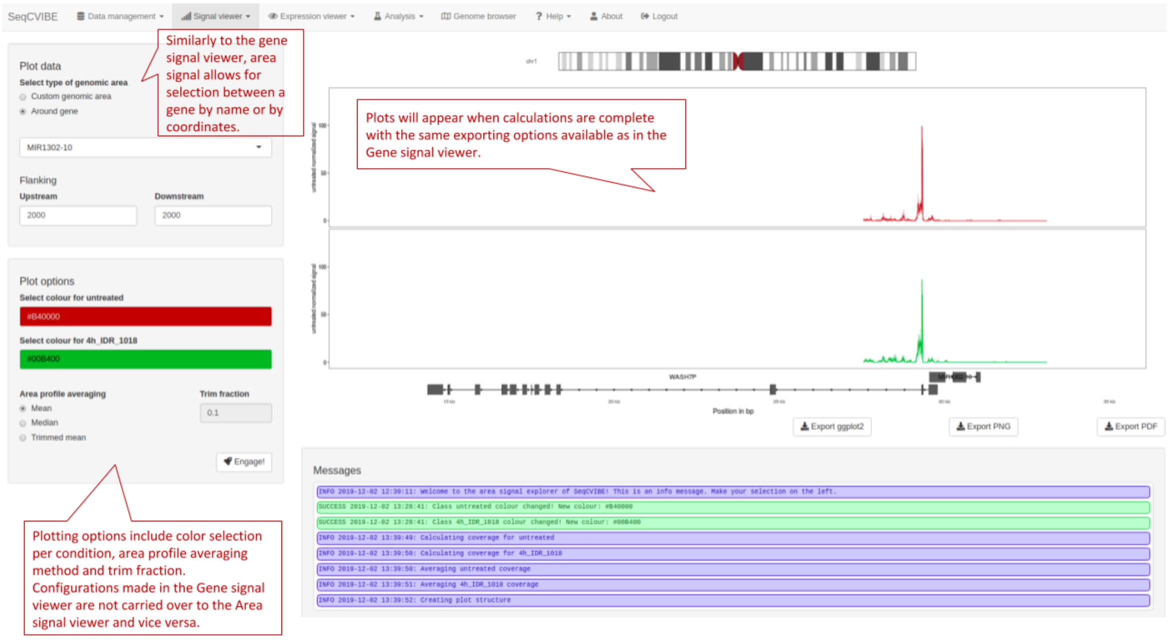

Figure S7: Creating RNA signal profiles over large genomic areas on the same chromosome.

2.2.1. Custom genomic area

Custom genomic area will visualize a chromosomal region of interest which is provided by the user. By default, known genes overlapping the given area will be automatically detected. However, if the user wants any specific regions of interest to be also included to the automatic detection, along with the known genes, the user will have to check the option ‘Include custom transcribed areas’ and provide the desired regions either by choosing to include the gene explorer entries, or by manually inserting the custom regions.

2.2.2. Around gene area

This option resembles the 'Gene signal' to a great extend as it plots the requested genomic area that is centered around a specific gene of interest. The gene can be selected from the drop-down menu right below, while the upstream and downstream limits of the plot can be given from the 'Flanking' options bars

2.3. Plot options

Plot options are to a large extend identical between Gene signal and Area signal plotting. First option is the flanking region limits. Presets are 2000 base-pairs upstream and downstream the region of interest. Other plotting options include selecting a color for every condition of the experiment and finally, selecting a method to be used in the profile averaging of the area to-be-plotted. These methods include Mean signal, Signal median and the trimmed mean. In the latter case the user can select the fraction to be trimmed manually. Here, it is worth noting that these options are relevant to their own tab selection and are therefore separate between area and gene signal plotting.

2.4. Exporting plots

There are currently three ways a user can export a plot. The **Export ggplot2** method exports a .rda file. This file can be loaded in R and allow amore experienced user to further modify the exported visualization. Alternative exporting options are in the **PNG** and **PDF** file formats. All exports are stored by default in the system's dedicated 'Downloads' directory. These exporting options are common across all other plot panels.

3. Expression viewer

The expression viewer tab is a presentation of the features (genes/transcripts) counts table, which is the backbone of a differential expression analysis in an RNA-Seq experiment. The table demonstrates the expression measurements for the input selection of genes or genomic regions. The number of tables to be displayed depends on the number of experimental conditions of the dataset under exploration. Therefore, if a dataset has two conditions, e.g. wild-type and disease, the top table will display wild-type expressions, while the bottom table, disease gene expressions.

The first column of the table is the name of the gene or the selected region, the second is the mean (by default) averaged expression and the third is the expression deviation between samples. Default deviation measure is the standard deviation. If the experiment contains more than two conditions, an equivalent number of additional tables will be displayed.

3.1. Known genes expression

The first option of the ‘Expression viewer’ is the 'Known genes' tab. This will allow the user to obtain a table measuring expression of a single gene, a custom set of genes, or even all genes of the studied organism. The input genes can be selected either from the given list of known gene names of the organism, or as a custom gene name input, where every entry is separated by a new line. Finally, there is also the choice of viewing expression for all genes, if the third option is clicked.

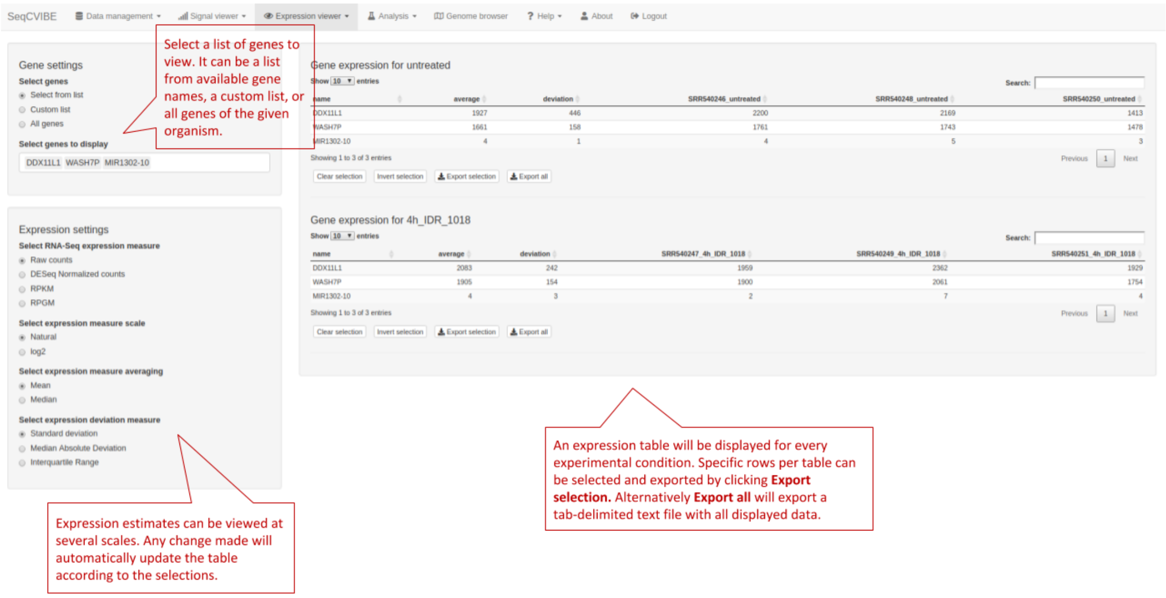

Figure S8: Viewing the expression of all or specific genes for all conditions of a created dataset in various formats and scales.

3.2. Expression calculator

The expression calculator works the exact same way as the 'Known genes', the only difference being that the user needs to give a pre-defined genomic region as input. There are two ways this can be done. Either with the gene explorer or the custom regions. Genomic regions given to the gene explorer will be used to produce the expression tables in the first scenario. Alternatively, if the user selects 'Custom regions' an input area will appear right below where they can input the genomic coordinates they wish to inspect and click the 'Add' button. All added entries will appear in the mini-table below. Hitting the **Engage** button will generate the requested tables.

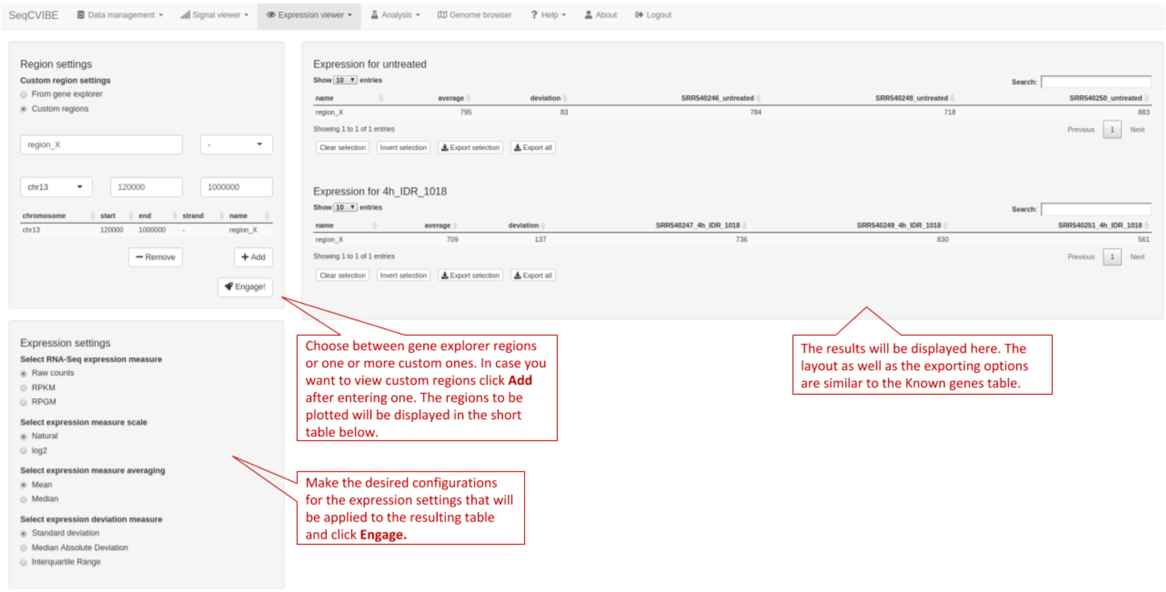

Figure S9: Calculating RNA abundance over custom, non-annotated regions.

3.3. Expression settings

There are a number of configurable parameters the user can tune to create a desired output expression table. The first one has to do with how the RNA-Seq expression will be measured. There are three available options:

- Raw and DESeq normalized read counts. This is the raw number of gene/genomic area read counts in each sample and conditions
- RPKM is the Reads per Kilobase per Million reads, a common abundance measurement in RNA-Seq
- RPGM refers to the DESeq normalized read counts divided by the sum of exon lengths for each gene

The second option is a scaling setting for the expression measure. The user gets to select between Natural and $\log_2$ scale. Log_2 scale is usually preferable as it models proportional changes rather than additive changes. This is typically biologically more relevant. The next option is the selection of the expression measure averaging method. Available options are 'Mean' and 'Median' Lastly, there is the expression deviation measure selection. Here the user can select among three methods by which the deviation of expression between the samples will be measured: Standard deviation, Mean absolute deviation and Interquartile range.

3.4. Exporting table

The resulting table can be exported in different ways. Rows of the resulting tables can be selected by clicking on them. Buttons below each table control what the user can do with the selection. 'Clear selection' removes all selected rows from the final selection. 'Invert selection' will include all non-selected rows to the final selection. When selection set has been finalized the user can click on **Export selection** to obtain a text tab-delimited file with all the requested information. In any case, the option **Export all** allows the user to export the whole dataset of gene expression. Exported tables and their settings are unique per condition.

4. Analysis

The analysis tab offers a toolkit for real-time analysis and visualization of an RNA-Seq data dataset. The four tools for analysis, visualization and exploration provided currently include Differential expression, Clustering analysis, Correlation analysis and Multidimensional scaling/Principal Component Analysis (MDS/PCA).

4.1. Differential Expression analysis

In the Differential expression tab the normalized read count data are used to perform statistical analysis to discover statistically significant changes in the abundance of expression levels between experimental groups. For example, in a typical experiment statistical testing would be used to decide whether, for a given gene, an observed difference in read counts is significant, that is, whether it is greater than what would be expected just due to natural random variation.

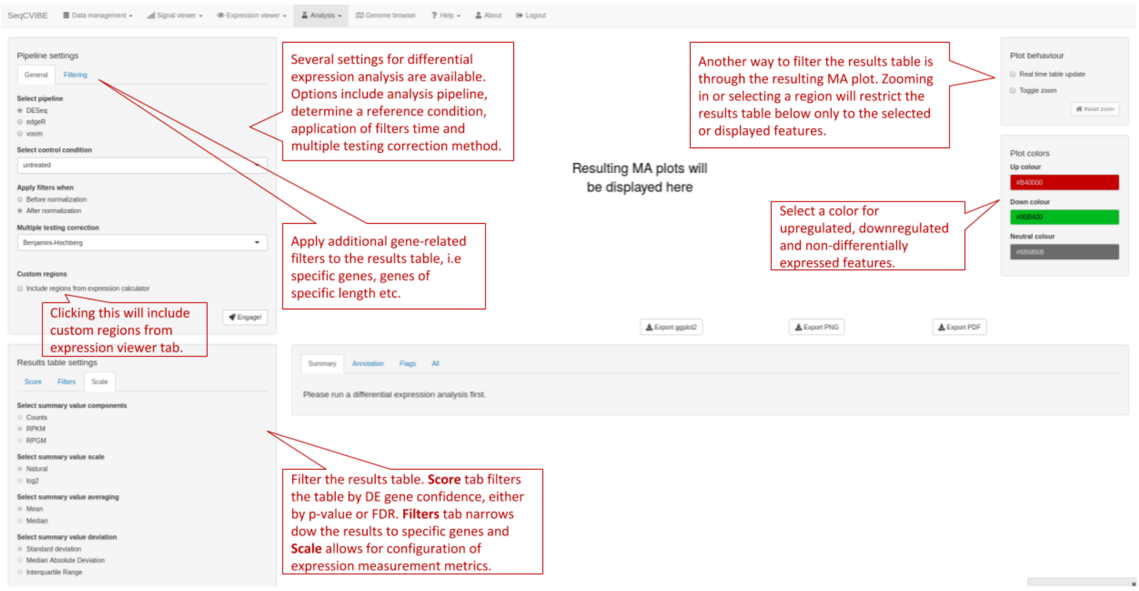

Figure S10: Defining the parameters for differential gene expression analysis.

4.1.1. Pipeline settings

General settings of the analysis include the following:

- The analysis pipeline that will be used. This only changes the tool that runs the analysis, which means there is a slight differentiation in the algorithm. Three widely used and fast enough for real-time analysis tools are included in the available options: DESeq, edgeR and voom.
- The condition of the experiment that will be used as control for the analysis. This can be selected from a drop-down menu.
- The multiple testing correction method, applied to correct p-values for multiple statistical testing.
- When the selected filters (next tab) will be applied to the results (before or after the normalization process).
- The inclusion of the custom regions from the expression calculator to the analysis results. If the tick box is clicked the results will include expression measurements for the custom regions.

Filtering settings, on the tab on the right will be applied to the analysis after it is complete, or beforehand, depending on the user's choice in the General settings tab. Basic gene filter options include:

- Mean/Median expression, quantile and known genes. If quantile gene filter is selected user will be prompted to fill in the desired percentile which will be used to filter genes.
- 'Known genes', where a drop-down list will appear where the user can select a set of genes whose expression will be averaged and used as a filtering cutoff (e.g. a set of genes known not to be expressed in the experimental setting investigated and used as noise estimation).
- Filtering a gene by its length in base-pairs .
- Filtering a gene based on its biotype.

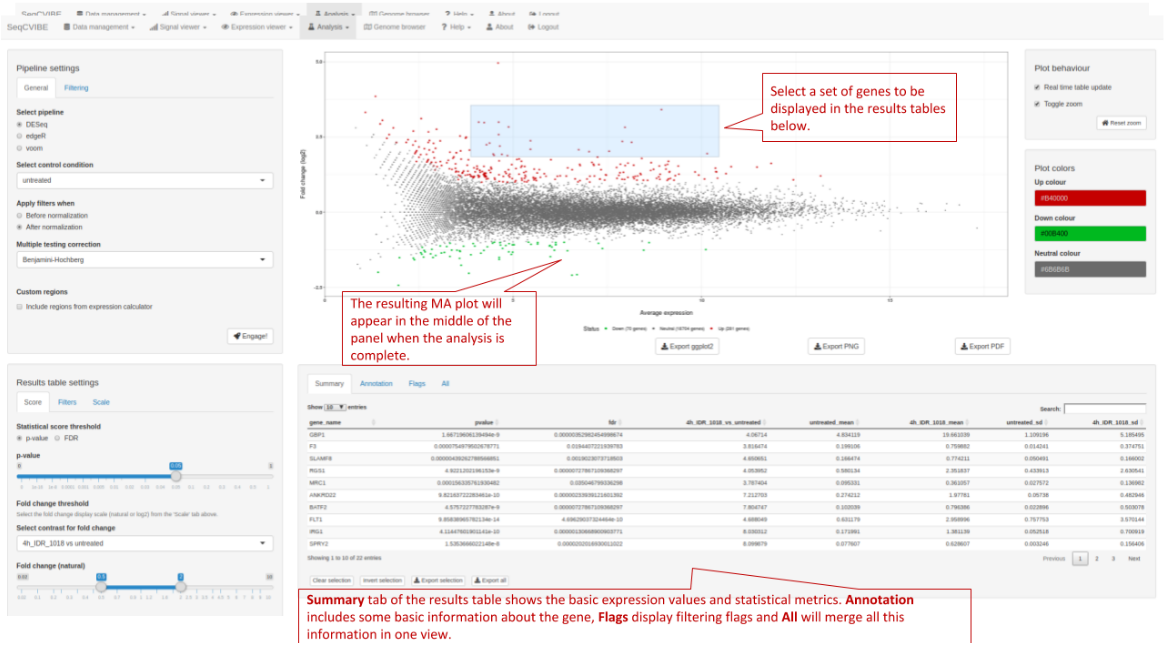

Figure S11: Exploring the results of differential gene expression analysis.

4.1.2. Results table

When the analysis is complete a Mean-Difference (or MA) plot will appear in the central panel of the differential expression analysis tab along with the results table right below. The table contains four tabs:

- Summary, which contains basic annotation elements and summary outcomes of the analysis for each gene. It consists of the following columns: gene name, the p-value of the findings for that gene, the false discovery rate, the expression ratio between different conditions of the experiment and lastly the mean and standard deviation counts for each condition.
- Annotation, which provides a table with the genomic coordinates of each differentially expressed gene, several annotation elements and its biotype.
- Flags, which contains 0 or 1 depending on whether the gene has passed a specific filter or not (filter flag names in columns).
- The last tab provides the option of combining all the aforementioned tables in one. Results table can be further configured with the 'Results table settings' panel. The user can control which significant gene hits will show up by choosing a threshold for one of the following measures: p-value, FDR or fold change. Post analysis filtering is available on the 'Filters' tab which is next. A mix of gene name, chromosome and biotype filters can be applied to further narrow down the results to something more meaningful to the user. Scale tab will allow for configuration of the measures used in the results table. By default, summary value components will be the gene counts, however RPKM and RPGM are also available. Similarly, the user might switch between natural and log2 count for the differential expression ratio, mean or median for averaging and

standard deviations, median absolute deviation or interquartile range for intra-sample deviation. The table can be fully or selectively exported, just like with the Expression viewer table.

4.1.3. Plot options

The resulting MA plot will appear after the analysis is complete. If real time table update is ticked, selecting an area of the plot will filter the results table only to the genes of interest contained in that area. By default gray spots indicate a gene that is not differentially expressed, red ones indicate an up-regulated gene while green dots indicate downregulated genes. Coloring options are available for all three categories in the 'Plot colors' panel.

4.2. Clustering analysis

Clustering analysis is a non-supervised classification algorithm that aims to group the set of samples included in the selected dataset in order to create clusters of similar samples which are dissimilar to samples in other clusters. It helps visualizing the intra-sample diversity of the datasets and helps determine if each of the experiment conditions are separated in well-differentiated categories. Clustering settings allow for the customization of the resulting heatmap.

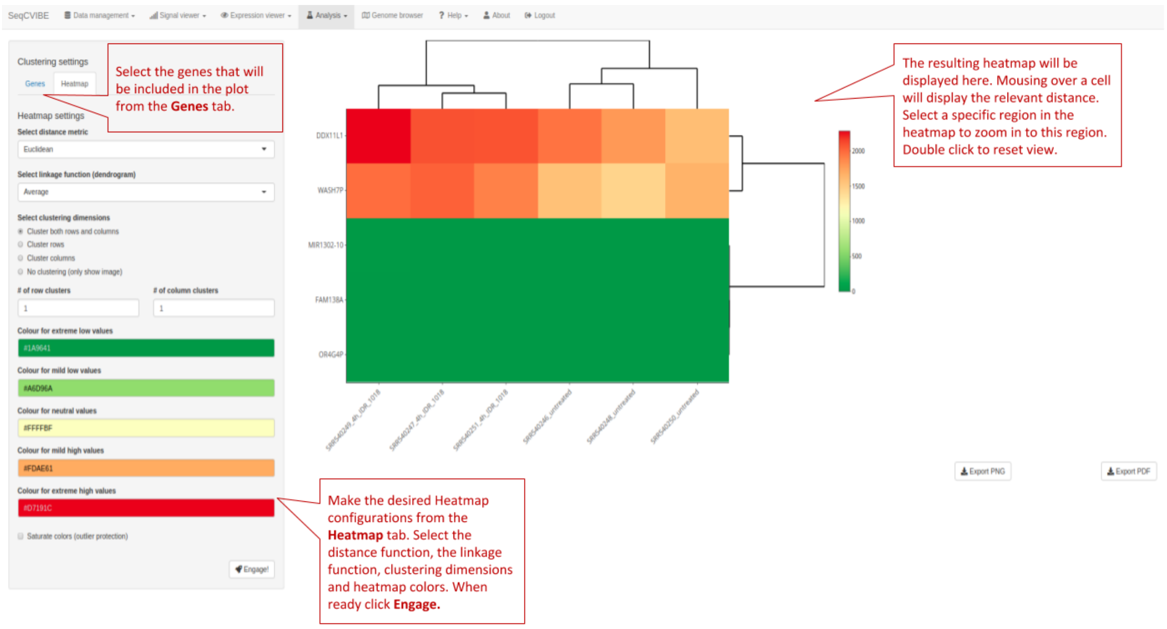

Figure S12: Performing cluster analysis of specific or differentialy expressed genes.

4.2.1. Gene settings

The gene settings tab allows the user to control which genes will be used to produce the heatmap. They can either be custom genes/regions or a specified gene list or just the genes that were found to be differentially expressed (if a differential expression analysis has already run). Next option is the

clustering variable selection. The user has to choose one of the available variables that will be used to define the clusters of the analysis. An expression measurement option is also available here, allowing for normalized counts, RPKM, RPGM measure in natural or $\log_2$ expression to be used for clustering.

4.2.2. Heatmap settings

Heatmap settings determine the mathematical parameters of the visualization. The first choice defines the metric that will be used to define the clusters distance. Default option is the Euclidean distance. Right below the user gets to select the linkage function that will be used to provide the relevant dendrogram of the resulting clusters. The default here is Average linkage. The clustering dimensions can also be modified right below along with the heatmap colors.

4.3. Correlation analysis

Correlation analysis will give an insight on how strong the relation is between samples included in the dataset in terms of their specific gene expression measurements or between gene expression levels among samples.

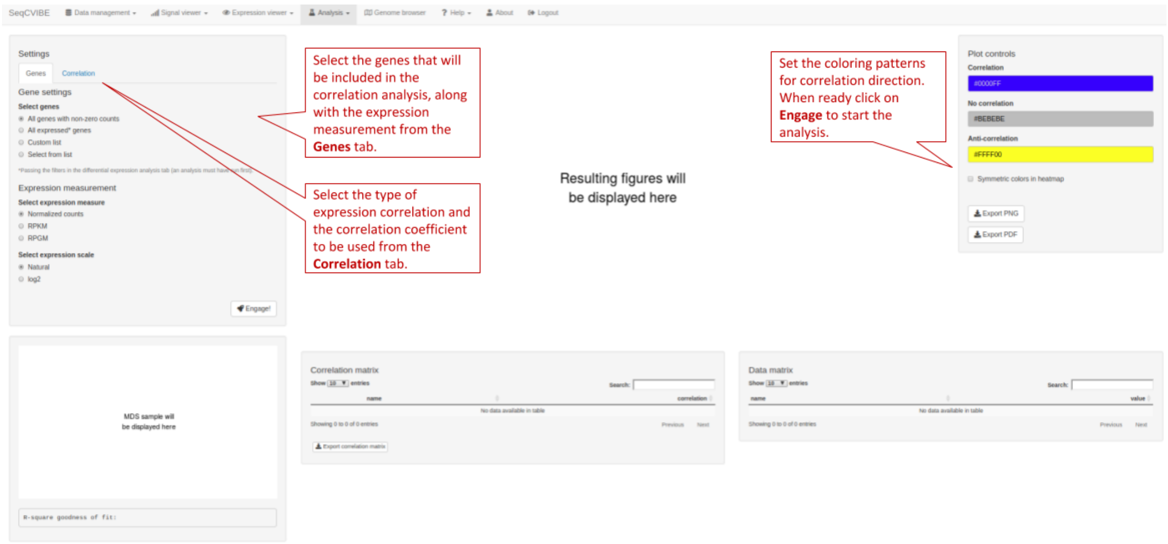

Figure S13: Options for gene expression correlation analysis.

4.3.1. Settings

There are several options to tune correlation analysis.

- Set of expressed genes will be used to calculate the correlation metric. The user can select all genes with non-zero counts, which is usually not feasible due to the large number of genes that will delay the calculations significantly, all expressed genes, which requires a differential

expression analysis to have already run, a custom gene list, or a custom selection of genes from the available drop-down box.

- Expression measurement settings will affect how the counts will be measured in the 'Data matrix' table below the correlation plot (see additional matrices).
- Correlation settings will determine the type of the expression correlation. 'Sample-wise' will correlate all samples based on their overall gene expression levels, 'Gene-wise' will correlate all genes given in the input list provided and 'Reference gene-wise' will calculate the correlation based on a given reference gene of interest.
- The correlation method can be selected next and can either be Pearson's or Spearman's.
- The colors for the resulting correlation heatmap selection panel.

Plots can be exported in PNG or PDF format.

4.3.2. Additional matrices

Right under the main correlation plot three additional supporting visualizations will be displayed after a successfully complete correlation analysis. From left to right there's the MDS plot giving a quick look on how well differentiated the samples are based on the genes selected for correlation. The middle table is the correlation matrix which shows the relevant correlation measures for the selected samples. The last table is more of a supporting data matrix which demonstrates the gene expression measures for the selected genes.

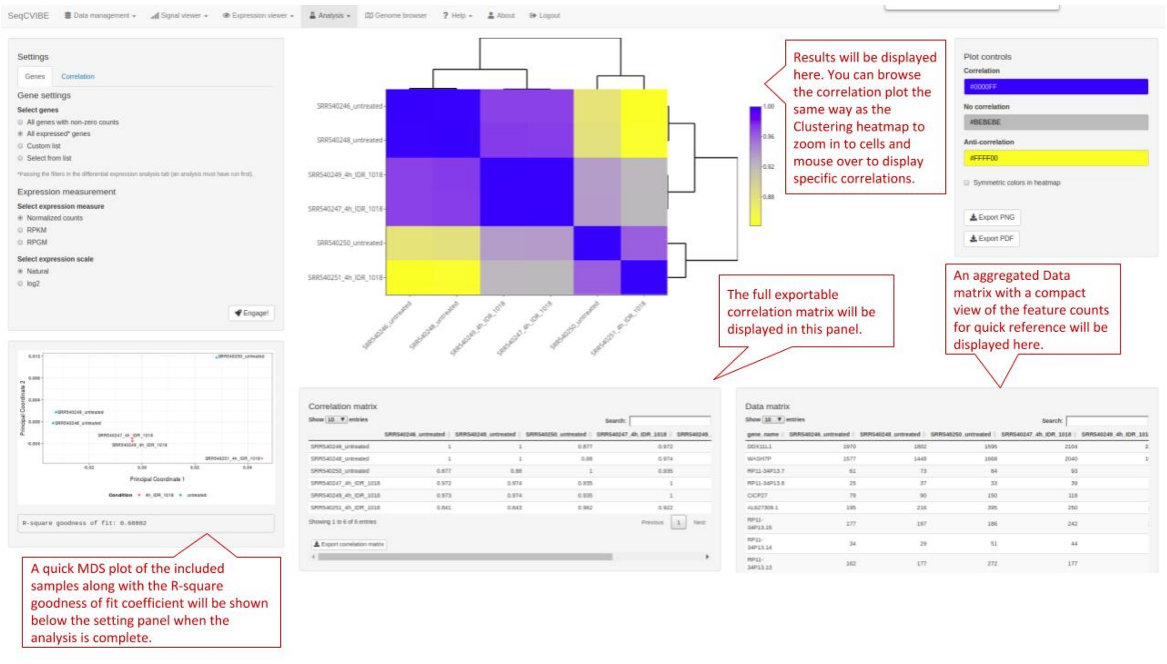

Figure S14: Exploring the results of gene correlation analysis.

4.4. MDS/PCA Analysis

The goal of MDS Analysis is to detect meaningful underlying dimensions that allow the user to explain observed similarities or dissimilarities (distances) between the investigated samples. Principal component analysis (PCA) is one of the commonly used statistical technique for finding patterns in data of high dimensions and expressing the data in such a way as to highlight their similarities and differences.

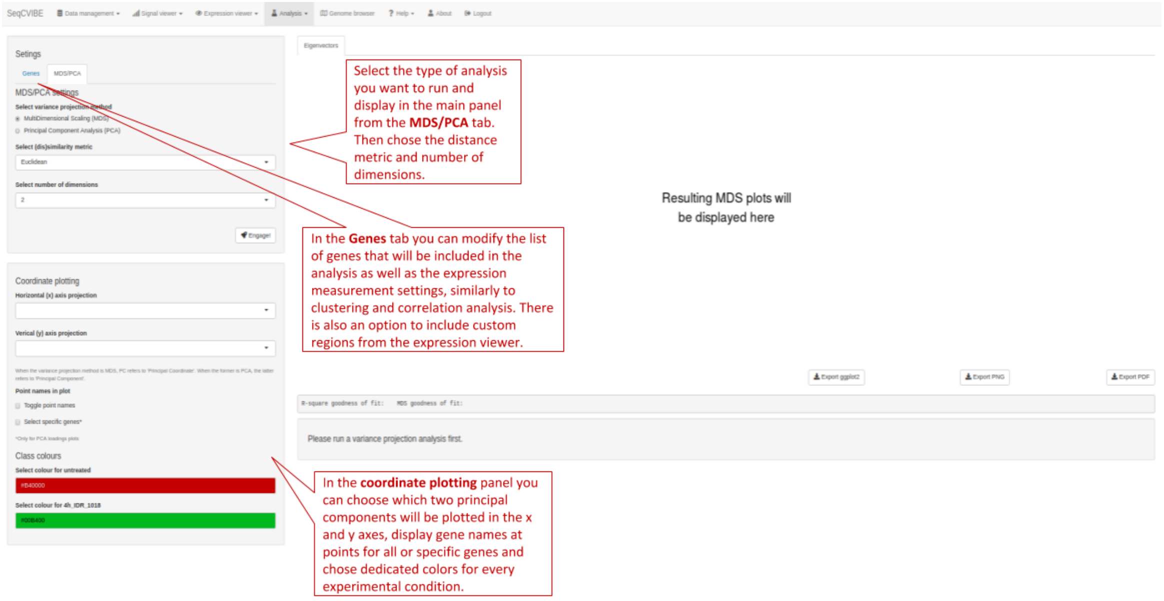

Figure S15: Options for MDS/PCA analysis.

4.5. Settings

Gene settings here, as in previous analysis tabs, are related to gene set selection and what expression measure and scale will be used for determining the intra-sample distances and patterns. 'MDS/PCA' tab sets the variance projection method that will be used between the two. Based on this selection, the relevant settings will appear right below. MDS settings allow for the selection of the (dis)similarity metric and the number of dimensions to be included. PCA settings allow selecting whether to scale the values or center them. Apart from PNG or PDF, the resulting plots can be exported as R objects as well.

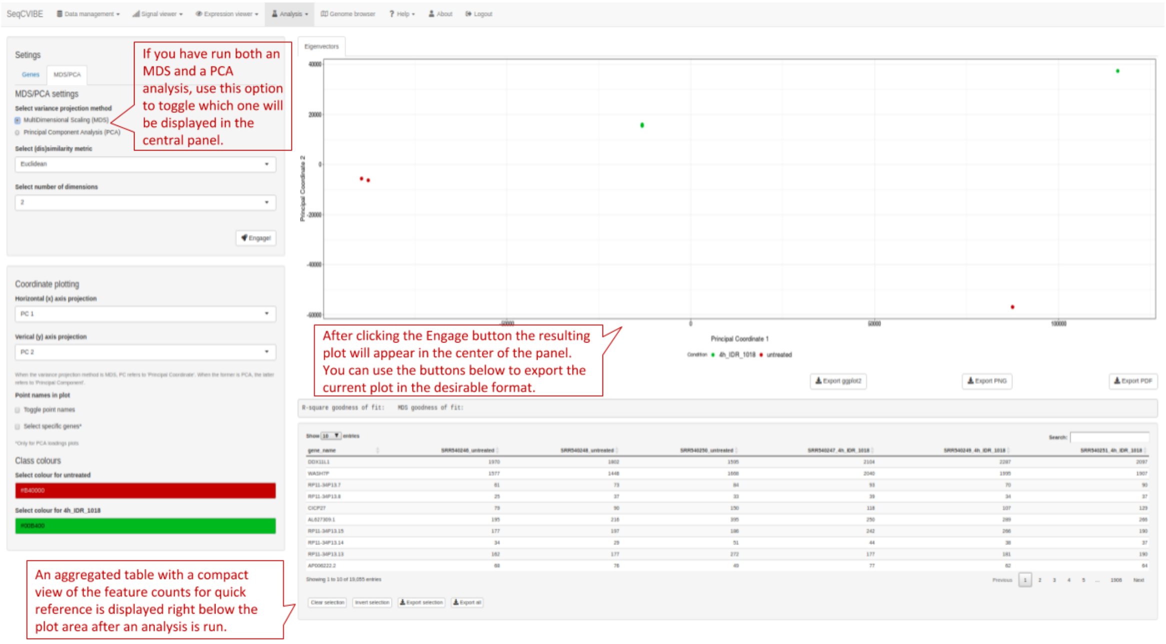

Figure S16: Exploring the results of MDS analysis.

4.6. Coordinate plotting

Coordinate plotting sets which two principal components or coordinates will be used in the plot and in which axis. Additional settings further down enable point names in the resulting plot or highlighting of any set of genes that might be of interest.

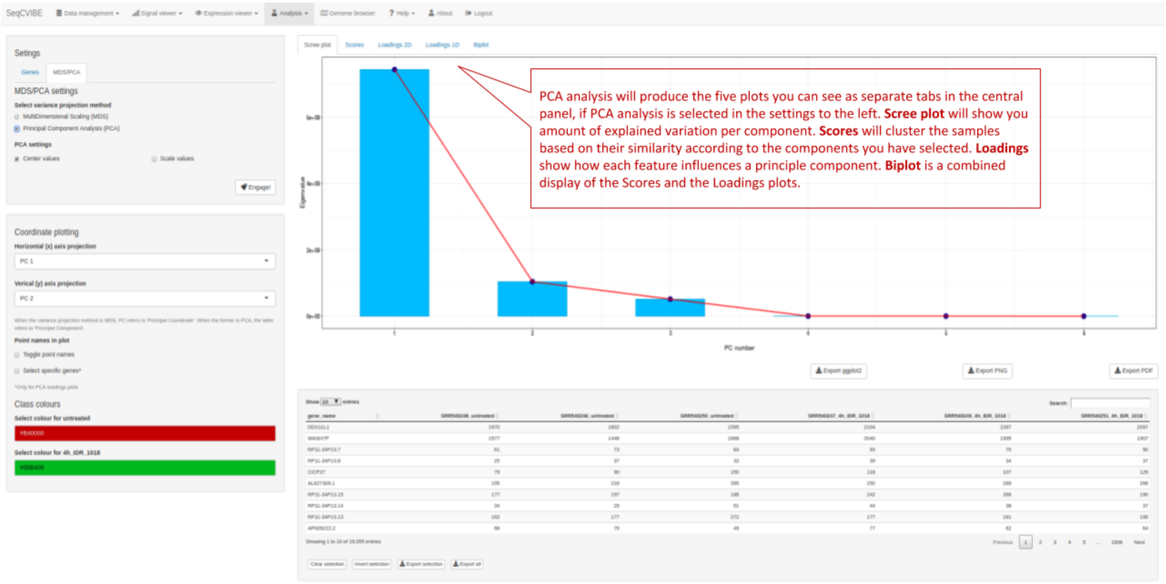

Figure S17: Exploring the results of PCA analysis.

5. Genome browser

Genome browsers are graphical tools that allow the display of information from biological databases, laid along linear representations of whole genomes. Apart from genomic features such as genes, gene predictions, gene expression atlases and many other types that can be depicted in layers across linear representations of chromosomes, genome browsers are ideal for depicting the actual signal derived from Next Generation Sequencing experiments.

Detailed RNA-Seq signal visualization across the genomes is achieved through the integration of JBrowse[1], a fast, embeddable in web applications genome browser. It is accessible for a created dataset through the **Genome browser** tab.

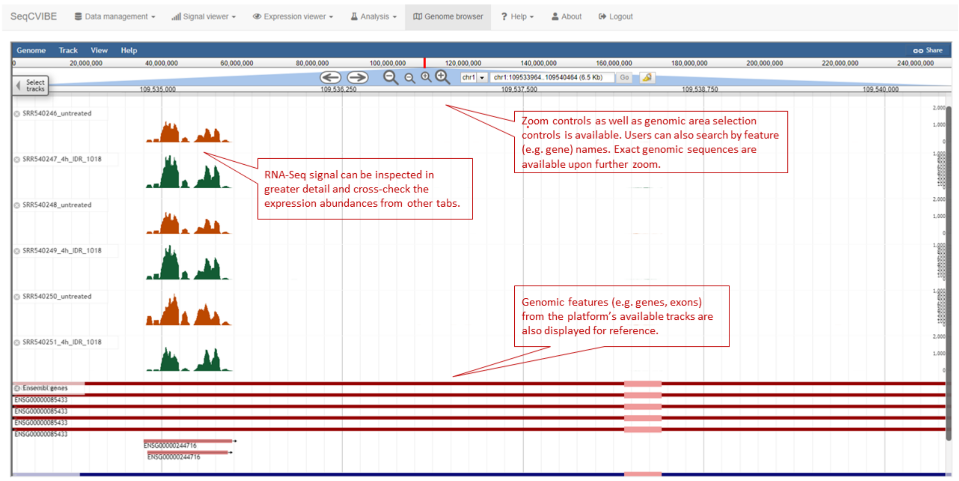

Figure S18: Inspecting and exploring RNA-Seq signals in depth through the integrated genome browser.

[1] http://jbrowse.org/

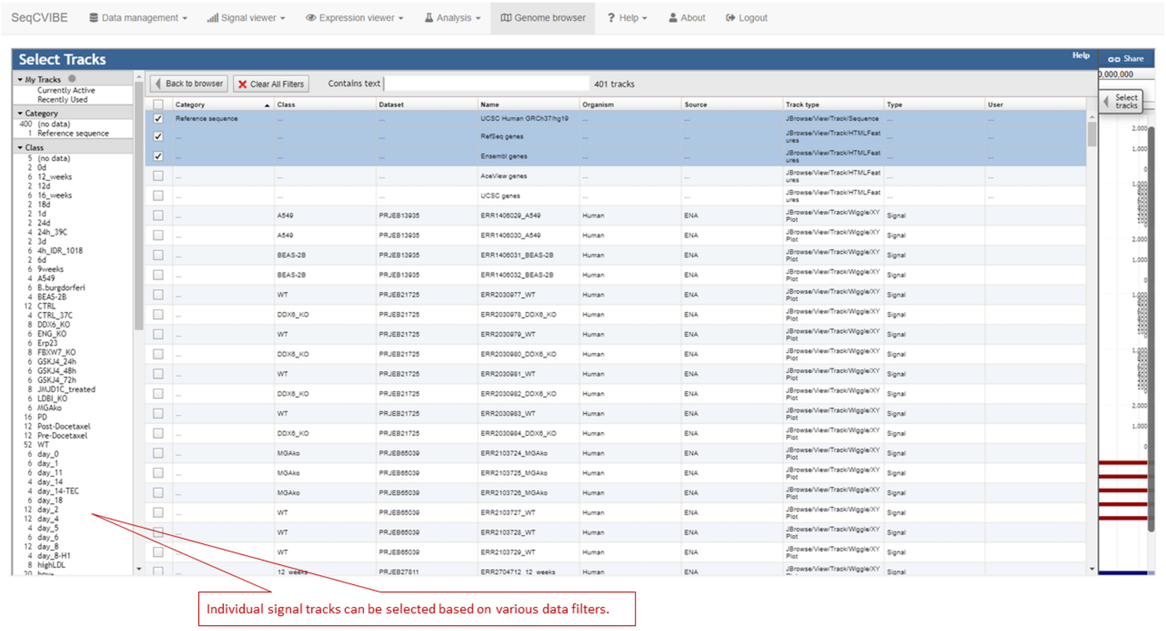

Figure S19: Selecting genome browser tracks to view.
